# Effect of Melittin on Metabolomic Profile and Cytokine Production in PMA-Differentiated THP-1 Cells

**DOI:** 10.3390/vaccines6040072

**Published:** 2018-10-13

**Authors:** Abdulmalik M. Alqarni, Valerie A. Ferro, John A. Parkinson, Mark J. Dufton, David G. Watson

**Affiliations:** 1 Strathclyde Institute of Pharmacy and Biomedical Sciences, University of Strathclyde, 161 Cathedral Street, Glasgow G4 0RE, UK; v.a.ferro@strath.ac.uk (V.A.F.); d.g.watson@strath.ac.uk (D.G.W.); 2 WestCHEM Department of Pure and Applied Chemistry, University of Strathclyde, 295 Cathedral Street, Glasgow G1 1XL, UK; john.parkinson@strath.ac.uk (J.A.P.); mark.dufton@strath.ac.uk (M.J.D.)

**Keywords:** melittin, pro- and anti-inflammatory cytokines, LPS stimulation, THP-1 cells, PMA differentiated, macrophages, adjuvant vaccine

## Abstract

Melittin, the major active peptide of honeybee venom (BV), has potential for use in adjuvant immunotherapy. The immune system response to different stimuli depends on the secretion of different metabolites from macrophages. One potent stimulus is lipopolysaccharide (LPS), a component isolated from gram-negative bacteria, which induces the secretion of pro-inflammatory cytokines in macrophage cell cultures. This secretion is amplified when LPS is combined with melittin. In the present study, pure melittin was isolated from whole BV by flash chromatography to obtain pure melittin. The ability of melittin to enhance the release of tumour necrosis factor-α (TNF-α), Interleukin (IL-1β, IL-6, and IL-10) cytokines from a macrophage cell line (THP-1) was then assessed. The response to melittin and LPS, applied alone or in combination, was characterised by metabolic profiling, and the metabolomics results were used to evaluate the potential of melittin as an immune adjuvant therapy. The addition of melittin enhanced the release of inflammatory cytokines induced by LPS. Effective chromatographic separation of metabolites was obtained by liquid chromatography-mass spectrometry (LC-MS) using a ZIC-pHILIC column and an ACE C4 column. The levels of 108 polar and non-polar metabolites were significantly changed (*p* ˂ 0.05) following cell activation by the combination of LPS and melittin when compared to untreated control cells. Overall, the findings of this study suggested that melittin might have a potential application as a vaccine adjuvant.

## 1. Introduction

Correct recognition of inflammatory signals in the body is vital to avoid the risk of autoimmune and chronic inflammatory diseases. Many of these signals arise from macrophages, and thus determination and investigation of the composition of local tissue microenvironments (e.g., metabolites, cytokines, and inflammatory signals) can be performed in order to understand the metabolic behaviour of macrophages following their activation [1]. Inflammation is a primary process that is triggered as a protective response to any stimulus of the innate immune system, such as injuries or infections, which pose a real threat to normal cell biology [2]. Inflammation can be initiated by first-line macrophage cells and dendritic cells to protect the body from external pathogens. Pathogen-associated molecular patterns (PAMPs) present in the invaders are recognised by specific receptors, the pattern recognition receptors (PRRs), present on the surface of these cells. PRRs play a crucial role in the stimulation of the innate immune system and the resulting signalling cascade [3]. The pathogen is targeted by the release of antimicrobial mediators, while further immune cells are recruited to the infection site by chemokine secretion. These chemokines include pro-inflammatory cytokines released by T and B lymphocytes to induce further inflammation and activate the adaptive immune response [4]. Several classes of PRRs have been characterised and investigated, including the toll-like receptors (TLRs) which are expressed on plasma membranes and activated by lipopolysaccharides (LPSs) from gram-negative bacteria [5].

The biosynthesis and/or depletion of cellular metabolites in response to pathogen invasion results in rapid alteration in the cellular metabolome [6]. Upon activation, innate immune cells undergo metabolic changes that can be described as similar to those that occur in cancer cells (Warburg effect) [4]. In the presence of oxygen, the metabolic profile of a tumour shows an increase in glycolysis under normoxic conditions, so that metabolism is shifted to produce lactic acid from pyruvate instead of the acetyl-CoA needed to feed the tricarboxylic acid (TCA) cycle and oxidative phosphorylation (OXPHOS) to generate adenosine triphosphate (ATP) [7]. The consumption of glucose and oxygen has long been known to increase in response to activation of TLR4 in innate immune cells by LPS. However, this oxygen consumption also reflects an increased generation of reactive oxygen species (ROS) [8]. Some enzymes in the glycolysis pathway, such as hexokinase and glucose-6-phosphate dehydrogenase, also show increased expression, leading to a further increase in glycolytic activities [9]. Inflammatory macrophages, when induced with LPS to release pro-inflammatory cytokines, therefore show a decreased utilisation of both the TCA cycle and OXPHOS metabolites as a consequence of this increase in glycolysis (glucose to lactate) to compensate the lack of ATP production via oxidative phosphorylation [10,11]. Interestingly, the cell metabolite adaptation occurs even in the presence of “aerobic glycolysis” [12]. This metabolic adaptation works as a survival response that serves to maintain mitochondrial membrane potential and cell integrity [13].

Understanding the process of macrophage activation by LPS is important for the identification of suitable immune stimulators, and these metabolic shifts could therefore be useful in this type of research. Macrophages stimulated by LPS generally show one or more of the following main metabolic shifts [4], which result in the expression of specific sets of genes. The first is a highly increased expression of inducible nitric oxide synthase (iNOS), which leads to the generation of citrulline and nitric oxide (NO) [14]. The latter results in the process of iron–sulphur protein nitrosylation in the electron transport chain which decreases mitochondrial respiration by inhibiting OXPHOS [15]. The second shift occurs due to activation of the glycolysis pathway in macrophages through activation of hypoxia inducible factor-1α (HIF-1α) [4], which then promotes the expression of its target genes, such as GLUT1 [16] and lactate dehydrogenase [17]. The increase in HIF-1α expression can be achieved by the activation of the mammalian target of rapamycin (mTOR), which is promoted by the translation of mRNAs with 5’TOP (5’-terminal oligopyrimidine) [18]. The third shift arises by an increase in the expression of phosphofructokinase-2 (PFK2) isoforms (i.e., u-PFK2), which causes an increase in the level of fructose-2,6-bisphosphate (F-2,6-BP) and activates the 6-phosphofructo-1-kinase glycolytic enzyme [19]. The fourth shift is due to inactivation of adenosine monophosphate-activated protein kinase (AMPK) in macrophages, which causes a decrease in fatty acid β-oxidation and mitochondrial metabolism. These actions boost the biosynthesis of inflammatory mediators and inhibit the catabolic pathways of the AMPK enzyme [20].

One promising immune modulator is melittin, the major lytic peptide of bee venom (BV). Many compounds have been isolated from BV, but melittin is the major component, accounting for as much as 50–60% of dried whole BV. Other components include peptides and proteins such as apamine, mast cell degranulating (MCD) peptide, secapin, adolapin, phospholipase A2, and hyaluronidase [21]. The enzymes, though present in relatively small amounts, are notable for being powerful allergens, and in some cases (e.g., phospholipase A2) synergise directly with melittin in its lytic action. Melittin is a 26-mer peptide with a characteristic sequence that provides the peptide chain with amphiphilic detergent-like properties and a high positive charge. It can self-assemble into tetramers depending on conditions [22,23] and has attracted considerable attention for its potential for selective destruction of cancer cells [24,25].

Melittin shows antiviral, antifungal, antibacterial, and anti-parasitic properties according to several studies and has been proposed to act at cell membrane level [26,27,28]. However, previous results regarding the effect of melittin on the immune system are inconsistent, indicating a need to perform more investigations. For example, melittin was characterised as an inhibitor of the release of prostaglandin E2 (PGE2) and nitric oxide (NO) from LPS-treated RAW 264.7 cells and synoviocytes, which showed a dose dependent inhibition of LPS-induced Cyclooxygenase enzyme (COX-2) and iNOS when treated with BV and melittin [29]. Similarly, melittin treatment of Propionibacterium acnes (*P. acnes*)-induced THP-1 monocytes significantly inhibited production of the pro-inflammatory cytokines TNF-α and IL-1β and cleavage of caspase-2 and -8 [30]. The mechanism of this inhibition was believed to occur in one of two possible ways: by the inhibition of Nuclear factor-kappa B (NF-κb) activation by melittin through interaction with the p50 subunit or by phosphorylation of IκB subunit [29,31]. The NF-κB transcription factor is crucial in inflammation as the expression of most of the pro-inflammatory genes rely on activation by this factor [32]. Anti-inflammatory activity of melittin and its interaction with LPS was referred to its amino acid sequences, particularly hydrophobic leucine zipper sequence [33]. By contrast, Stuhlmeier reported that neither BV nor melittin blocked IL-1β induced activation of NF-κB or had any effect on IκB phosphorylation or degradation using electrophoretic mobility shift (EMSA) assay in several cell types [21]. Moreover, BV and melittin, even at high concentrations, did not compete for NF-κB-p50-DNA binding or show any interactions. However, the mRNA levels of many types of pro-inflammatory genes and COX-2 protein were significantly elevated following exposure to BV and melittin. In addition, oxygen radicals were released in large amounts in a dose-dependent manner in response to BV treatment [21]. Another study, which examined the effects of BV and its isolated compounds on the production of pro-inflammatory cytokines in phorbol 12-myristate 13-acetate (PMA)-differentiated U937 cells, showed that the expressions of TNF-α, IL-1β, and IL-6 cytokines increased in response to melittin and LPS co-stimulation when compared to LPS stimulation alone [34].

Melittin can also be used as an adsorption enhancer in Caco-2 cells [35], indicating its possible usefulness as an adjuvant for nasal administrations [36]. In addition, melittin has been reported to enhance antibody titres when co-administered with tetanus and diphtheria toxoid, which facilitated the longevity of the immune response when compared to antigen administration alone [37]. Based on these observations, melittin shows significant effects on cell inflammatory signalling, which support the use of melittin as an efficient immunomodulatory agent rather than for pro-inflammatory cytokine neutralisation. More investigations are needed to assess the differences in the mechanism of action of LPS and melittin, and the synergy between melittin and LPS in triggering the immune system. One possible strategy would be to evaluate the metabolic changes that occur in macrophages in response to melittin and LPS when administered singly or in combination.

Metabolomics is one way to provide a quantitative identification of endogenous and exogenous cell metabolites to reveal the relative pathway relationships between metabolites and observed physiological and/or pathological alterations. Metabolomics studies are now providing a number of advantages for determining health status, such as providing predictive powers and diagnosing the disease state, revealing biological markers for drug responses, and defining the links to genetic variants [38]. Most metabolomics studies use untargeted metabolomic profiling for analysis of small molecule metabolites and are typically performed by nuclear magnetic resonance (NMR) spectroscopy and mass spectrometry (MS), highly sensitive and accurate instruments. The coupling of MS to high performance liquid chromatography (HPLC) allows the separation and identification of thousands of metabolites in a single biological sample. Many research studies are now focusing on using metabolomics in diverse areas [24,39,40], including the study of inflammatory related metabolites [41,42]. For instance, Traves et al. characterised the metabolic network flux and changes and its relation to signal transduction in LPS-activated macrophages [43].

Effects on many metabolites have been described in observations of innate immune cell metabolism. Understanding the interface between these immune cells and their metabolism could therefore provide novel tools for manipulating cellular activities. For example, the production of pro-inflammatory cytokines contribute to systemic inflammation [44]. Similarly, the loss of cellular mass promoted by TNF-α has been characterised in cachexia, demonstrating a link between inflammation and metabolism [45]. The quality and the benefit-to-risk ratio of immunisations could be improved by identification of specific metabolites; for example, metabolic profiling of patients pre- and post-smallpox vaccination was able to distinguish patient clinical samples with myocarditis and asymptomatic elevation of troponins, suggesting the potential for identification of biomarkers related to adverse vaccine reactions [46,47]. Moreover, understanding alterations in metabolomics pathways due to vaccination could help to improve new target vaccine designs (adjuvants, antigens and nanoparticle carrier systems), as reported by Gray et al., who used Ultra high performance liquid chromatography-mass spectrometry (UPLC-MS) metabolomic profiling of plasma in calves following vaccination to determine the immune-correlated metabolite characteristics of immune responses [48]. The current knowledge of immune metabolism has been widely advanced by improvements in metabolomics-based technologies.

Major efforts are now focused on the identification and innovation of new pharmacological compounds to enhance immune responses. The aim of the current study was to use enzyme-linked immunosorbent assay (ELISA) to evaluate the changes in the production of pro- and anti-inflammatory cytokines (TNF-α, IL-1β, IL-6, and IL-10) following the administration of melittin to PMA-differentiated THP-1 cells. A secondary aim was to examine the changes in the metabolic profile of THP-1 macrophage cells in response to melittin in the presence and absence of LPSs. This was performed using an LC-MS-based metabolomics approach employing ZIC-pHILIC and ACE C4 columns for polar and non-polar metabolites, respectively. The determination of the metabolic changes in response to melittin in stimulated monocyte-derived macrophages cells could be of value for many vaccine applications and the main objective of the present study was to evaluate this potential.

## 2. Materials and Methods

### 2.1. Sample Isolation and Preparation

Melittin was isolated and purified from BV (supplied lyophilised by Beesen Co. Ltd., Dae Jeon, Korea) using reversed phase medium pressure liquid chromatography (MPLC) on a Reveleris^®^ iES flash chromatography system (Grace Davison Discovery Sciences, Carnforth, UK) with dual UV (λ = 220/280 nm) and evaporative light scattering detection (ELSD). This was performed using the same method previously described [49]. The resultant BV fractions were freeze-dried and stored at −20 °C until required for the assays.

### 2.2. Cell Culture and Differentiation

The THP-1 cell line was obtained from American Type Culture Collection-ATCC^®^ (Porton Down, Salisbury, UK) and maintained at a 1 × 10^5^ cell/mL seeding density in RPMI 1640 (Thermos Fisher Scientific, Loughborough, UK) containing 10% (*v/v*) foetal calf serum (FCS) (Life Tech, Paisley, UK), 2 mmol/L L-glutamine (LifeTech, Paisley, UK), and 100 IU/100 µg/mL penicillin/streptomycin (Life Tech, Paisley, UK). Cells were sub-cultured using fresh media every 2–4 days and maintained in an incubator (37 °C, 5% CO_2,_ 100% humidity). THP-1 cells were differentiated using PMA (Sigma-Aldrich, Dorest, UK) at a final concentration of 60 ng/mL and incubated for 48 h. THP-1 cell differentiation was enhanced by removing the PMA-containing media and adding fresh media for a further 24 h. Cells were checked under a light microscope for the evidence of differentiation.

### 2.3. Cell Viability Assay

The THP-1 cells were seeded at a density of 1 × 10^5^/well in 96-well plates and incubated for 24 h at 37 °C in a humidified atmosphere of 5% CO_2_. After 24 h, the cells were treated with different concentrations of melittin (0.39–100 µg/mL) and incubated for a further 24 h. Untreated control cells and medium were added to the plates and dimethyl sulphoxide (DMSO) was used as a positive control. Resazurin salt solution (0.1 mg/mL) was added at a final concentration of 10% (*v/v*) and the plates were incubated for a further 24 h. Fluorescence readings were taken using a SpectraMax M5 plate reader (Molecular Devices, Sunnyvale, CA, USA) at λ_Ex_ of 560 nm and λ_Em_ of 590 nm. After background correction, cell viability for each concentration was calculated relative to the mean value of negative control (*n* = 3). GraphPad Prism for Windows (version 5.00, GraphPad Software, San Diego, CA, USA) was used to obtain dose–response curves and mean inhibitory concentration (IC_50_) values.

### 2.4. Cytokine Production

After 48 h of differentiation using PMA (60 ng/mL) in 24-well plates, the media were aspirated, and the cells were incubated for a further 24 h in PMA-free medium. At day 4, the cells were incubated with final concentrations of melittin (0.5 and 1 µg/mL) with and without LPS (Sigma-Aldrich) (0.5 and 1 µg/mL) for an additional 24 h. Conditioned medium was collected and frozen until required for ELISA (*n* = 3).

### 2.5. Enzyme-Linked Immunosorbent Assay (ELISA)

ELISA Ready-Set-Go kits were purchased from Thermo Fisher Scientific (Loughborough, UK). The assays were performed according to the manufacturer’s instructions to quantify the release of inflammatory cytokines (TNF-α, IL-1β, IL-6, and IL-10). The reaction was stopped using acid solution (2 N sulphuric acid). The plates were read using a SpectraMax M5 plate reader (Molecular Devices, Sunnyvale, CA, USA) at 560 nm and the absorbance values were corrected by subtracting readings taken at 570 nm.

### 2.6. Metabolite Extraction

The PMA-differentiated THP-1 cells were grown for 48 h in 6-well plates seeded at a density of 4.5 × 10^5^/well (*n* = 6). The medium was aspirated and replaced with fresh medium for a further 24 h, and then the cells were incubated with LPS, melittin, or a combination of LPS and melittin for an additional 24 h. The final concentrations of LPS and melittin were 0.5 and 1 µg/mL, respectively. After 24 h, the medium was aspirated, and the cells were washed with 3 mL of phosphate-buffered saline (PBS) (Sigma-Aldrich) at 37 °C. The cells were extracted (1 mL per 1 × 10^6^ cells) by ice cold extraction solution (methanol:acetonitrile:water, 50:30:20 (*v/v*), containing 5 µg/mL of internal standard ^13^C_2_ glycine (Sigma-Aldrich, Poole, UK)). The cells were scraped, and cell lysates were mixed in a Thermomixer (12 min, 4 °C), and then centrifuged for 15 min at 0 °C (13,500 r.p.m.). The supernatants were collected and stored at −80 °C until required for LC-MS analysis. The stability and reproducibility of the analytical method was ensured by injecting authentic standard metabolite mixtures and quality control (QC) samples throughout the runs. The analytical standards were prepared by adding 10 µg/mL final concentration of each metabolite standard [50] containing ^13^C_2_ glycine, distributed into seven different standard solutions. The pooled quality control samples were prepared by pipetting 20 µL from each of the samples and mixing them together before transferring them into a HPLC vial. A mixture of fatty acid standards was prepared from a mixture of 37 fatty acid methyl ester standards supplied by Sigma Aldrich (Supelco 37 component FAME Mix) by hydrolysis with 1 M methanolic KOH followed by extraction into hexane.

### 2.7. LC-MS Conditions

An Accela HPLC system interfaced to an Exactive Orbitrap mass spectrometer (Thermo Fisher Scientific, Bremen, Germany) was used for the liquid chromatographic separations. ZIC-pHILIC (150 × 4.6 mm, 5 µm) and ACE C4 (150 × 3.0 mm, 3 µm) HPLC columns supplied by HiChrom (Reading, UK) were used. Samples were run on LC-MS under the following conditions: the ZIC-pHILIC mobile phase consisted of 20 mM ammonium carbonate in HPLC-grade water (A) and acetonitrile (B); the solvent gradient used was 80% B (0 min), 20% (30 min), 8% (31–36 min), and 80% (37–45 min) at a flow rate of 0.3 mL/min. For the ACE C4 column, the mobile phase was 1 mM acetic acid in water (A) and 1 mM acetic acid in acetonitrile (B). The solvent gradient used was 40% B (0 min), 100% (30–36 min) and 40% (37–41 min) at a flow rate of 0.4 mL/min. The nitrogen sheath and auxiliary gas flow rates were maintained at 50 and 17 arbitrary units. The electrospray ionisation (ESI) interface was employed in a positive/negative dual polarity mode, with a spray voltage of 4.5 kV for positive mode and 4.0 kV for negative mode, while the ion transfer capillary temperature was set at 275 °C. Full scan data were obtained in the mass-to-charge ratio (*m*/*z*) between 75 and 1200 amu for both ionisation modes. The data were collected and processed using Xcalibur 2.1.0 software (Thermo Fisher Scientific, Bremen, Germany).

### 2.8. Data Extraction and Statistical Analysis

The data were extracted using MZMatch software (SourceForge, La Jolla, CA, USA), http://mzmatch.sourceforge.net/). A macro-enabled Excel Ideom file was used to filter, compare and identify the metabolites (http://mzmatch.sourceforge.net/ideom.php). The metabolite lists obtained from these searches were then carefully evaluated manually by considering the quality of their peaks and the metabolites were matched with the retention times of authentic standards mixtures run in the same sequences. Library searches were also used for identification and carried out against accurate mass data of the metabolites in the Human Metabolome Data Base, KEGG (Kyoto Encyclopedia of Genes and Genomes), and lipid maps. All metabolites were within 3 ppm of their exact masses. Univariate comparisons were performed using Microsoft Excel and paired *t*-tests between treated and control cells and differences were considered significant at *p* < 0.05. SIMCA-P software v.14.0 (Umetrics, Umea, Sweden) was used for multivariate analysis of the metabolite data by fitting PCA-X and OPLS-DA.

## 3. Results

### 3.1. BV Fractionation and Isolation of Melittin

A crude BV sample was fractionated by MPLC into three major fractions (F-1, F-2 and F-3). As previously described [49], F-1 and F-2 were determined to contain mixed components, while F-3 was determined to contain essentially a single component, melittin (Figure S1).

### 3.2. Cytotoxicity of Melittin against Normal and PMA-Differentiated THP-1 Cells

**Figure 1:**
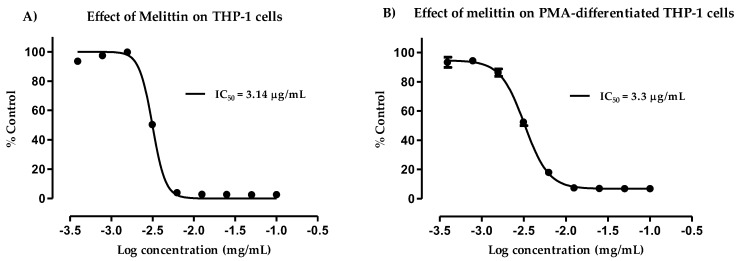
Cytotoxic effect of melittin on THP-1 cells (**A**) and phorbol 12-myristate 13-acetate (PMA)-differentiated THP-1 cells (**B**). The observed effect was determined following administration of varying doses of melittin. Melittin was cytotoxic to normal THP-1 and PMA-treated cells, with IC_50_ values of 3.14 and 3.3 µg/mL, respectively. The data represent the mean ± SD (*n* = 3).

Cytotoxicity assays were used to test the melittin isolated from BV. The studies were performed on THP-1 cell lines before and after differentiation with PMA to evaluate the differences in cell lysis due to melittin (Figure 1). Normal THP-1 cells were slightly more sensitive to melittin than the differentiated cells, according to their respective IC_50_ values of 3.14 and 3.3 µg/mL. Figure S2 shows a micrograph of the cells before and after PMA differentiation for 48 h. The monocyte-derived macrophages became adherent and showed a much greater increase in their cytoplasmic volume when compared to the untreated control cells [51]. In both cases, dose–response relationships were clearly observed. The cytokine production was analysed by ELISA following melittin administration at 0.5 and 1 µg/mL doses, whereas melittin was added at 1 µg/mL for the metabolomics study. The cells showed >90% viability at these melittin doses.

### 3.3. Effect of Melittin on the Production of Pro-Inflammatory TNF-α Cytokine

**Figure 2:**
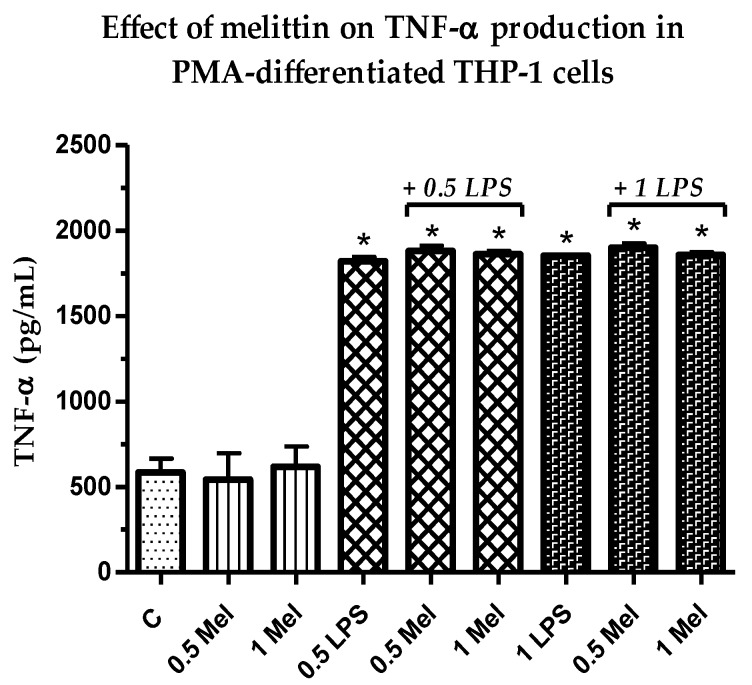
Effect of melittin on the production of tumour necrosis factor-α (TNF-α) cytokines in the presence and absence of LPS on PMA-differentiated THP-1 cells. All six treatments were significantly different from the negative control (*n* = 3). C: control; Mel: melittin; LPS: lipopolysaccharides; *: Significant (*p* < 0.05).

As shown in Figure 2, the concentrations of pro-inflammatory TNF-α in THP-1 derived macrophages were a slight to negligible effect by the combination treatments (LPS + melittin) when compared to the LPS positive control. The effects in cytokine production in response to LPS + melittin were not statistically significant (*p* > 0.05) from the LPS alone (Table S1); however, they were significant (*p* < 0.05) from the negative control cells. Two concentrations of melittin were assessed to examine dose dependency. Samples not treated with LPS showed a background level release of cytokine. The lack of effect of melittin on TNF-α production in LPS-stimulated macrophages was previously observed [52].

### 3.4. Effect of Melittin on the Production of Pro-Inflammatory IL-1β Cytokines

**Figure 3:**
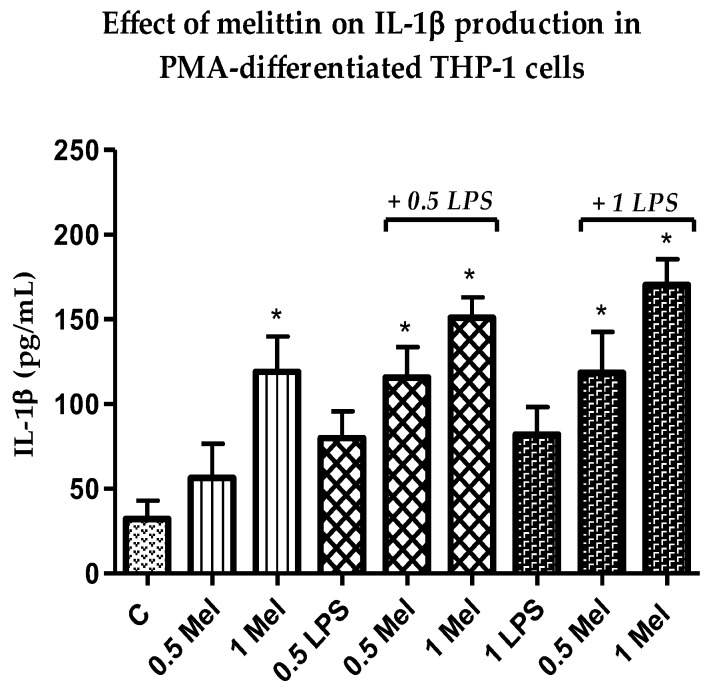
Effect of melittin on the production of Interleukin-1β (IL-1β) cytokines by PMA-differentiated THP-1 cells in the presence and absence of LPS. Cells treated with all four combination treatments showed significantly higher release when compared with negative control cells (*n* = 3). C: control; Mel: melittin; LPS: lipopolysaccharides; *: Significant (*p* < 0.05).

A clear increase was noted in the release of the pro-inflammatory IL-1β cytokine (Figure 3) in response to the combination of melittin and LPS when compared with LPS alone. This synergistic effect was statistically significant (*p* < 0.05) for melittin combinations in comparison with cells treated with LPS alone at 0.5 and 1 µg/mL when melittin was used at 1 µg/mL (Table S2). In contrast to TNF-α, secretion of LPS-induced IL-1β was induced dose-dependently in the THP-1-derived macrophages in response to melittin treatment. The maximum release of IL-1β was observed after the combined treatment with 1 µg/mL LPS and melittin. The release of IL-1β was also induced significantly by melittin alone when compared with the negative control cells [52].

### 3.5. Effect of Melittin on the Production of Pro-Inflammatory Il-6 Cytokines

**Figure 4:**
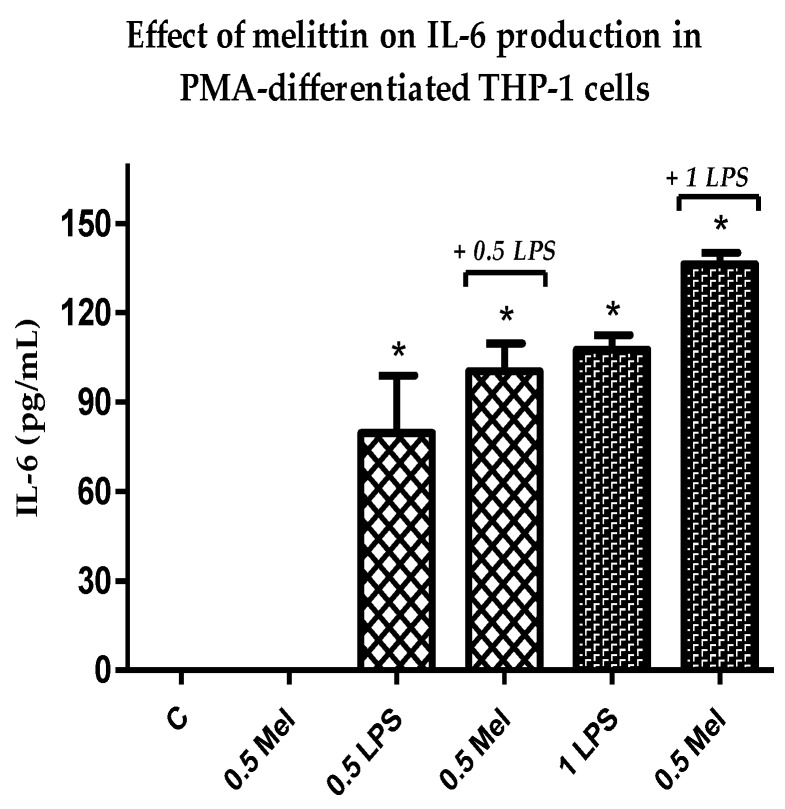
Effect of melittin on the production of IL-6 cytokine in the presence and absence of LPS on PMA-differentiated THP-1 cells. Melittin was tested at a 0.5 µg/mL final concentration in combination with LPS (0.5 and 1 µg/mL) (*n* = 3). C: control; Mel: melittin; LPS: lipopolysaccharide; *: Significant (*p* < 0.05) compared to negative control cells.

In agreement with the observed increase in the release of the pro-inflammatory cytokines above, IL-6 also showed an increase in production in response to treatment with a combination of melittin and LPS when compared to LPS alone. The effect was statistically significant (*p* < 0.05) for melittin and LPS combinations when compared with negative control cells and also with 1 µg/mL LPS alone (Table S3). Melittin effects were observed at a final concentration of 0.5 µg/mL, whereas effects were noted for two different doses of LPS (0.5 and 1 µg/mL). Maximum synergism was observed at a final concentration of 1 µg/mL and 0.5 µg/mL for LPS and melittin, respectively. Melittin alone had no effect on the level of release of this cytokine or the release was undetectable (Figure 4).

### 3.6. Effect of Melittin on the Production of Anti-Inflammatory Il-10 Cytokines

**Figure 5:**
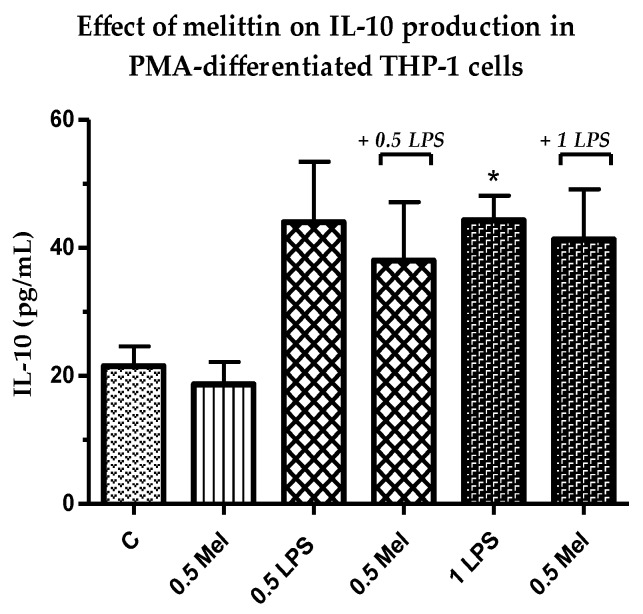
Effect of melittin on the production of IL-10 cytokine in the presence and absence of LPS on PMA-differentiated THP-1 cells. Melittin was tested at a 0.5 µg/mL final concentration in combination with LPS (0.5 and 1 µg/mL) (*n* = 3). C: control; Mel: melittin; LPS: lipopolysaccharide; *: Significant (*p* < 0.05).

The production of anti-inflammatory Il-10 was assessed using 0.5 µg/mL melittin. Interestingly, the release of this anti-inflammatory cytokine was decreased by the combination of melittin and LPS (0.5 and 1 µg/mL) when compared with LPS alone. However, the results of the combination treatments did not reach statistical significance. In addition, no effect was observed for cells treated with melittin alone when compared with the negative control cells (Figure 5).

### 3.7. Effect of Melittin on the Cell Metabolome

**Figure 6:**
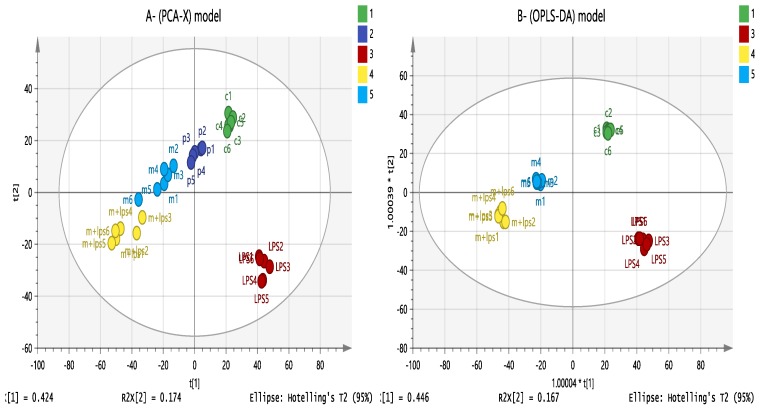
(**A**) PCA-X vs. (**B**) OPLS-DA score plots of THP-1 cells. The figures show a clear separation between control, pooled, and treatment groups (LPS, melittin, and LPS + melittin) based on 955 polar metabolites separated on a ZIC-pHILIC column (*n* = 6). PCA score plot (**A**) has R^2^X = 0.668, Q^2^ = 0.559. OPLS-DA score plot (**B**) has R^2^X = 0.744, R^2^Y = 0.99, Q^2^ = 0.903. (C: control; Mel: melittin; LPS: lipopolysaccharide; Mel + LPS: melittin and LPS combination treatments; *P* = pooled samples). PCA: principal component analysis.

Understanding how the metabolic response in monocyte-derived macrophages is activated by different stimuli has important clinical ramifications. The aim of the metabolomic profiling of PMA-differentiated THP-1 cells following melittin, LPS, and combination treatments was to identify the mechanism of action of melittin on macrophage cells and to try to understand its synergism with LPS in cytokine production. Figure 6A shows a clustering of quality control samples (P 1–4) in the middle of the plot, obtained using principal component analysis (PCA) of all the polar metabolites detected by analysis on the ZIC-pHILIC column. This finding validates the analysis and indicates good instrument stability and precision during the run. A clear separation was noted between the treatment and control groups using an OPLS-DA model (Figure 6B), which would suggest a different mechanism of action for the combination treatment (melittin + LPS) than for LPS or melittin alone.

**Figure 7:**
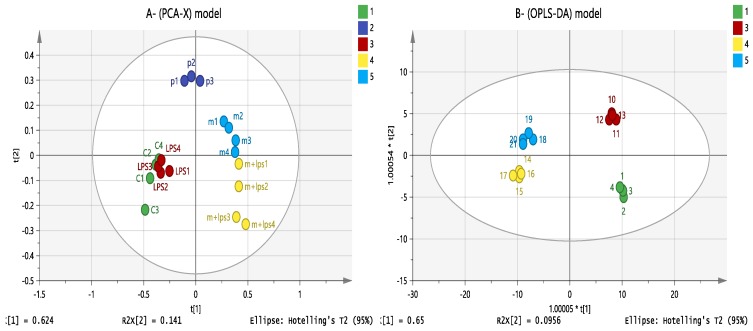
(**A**) PCA-X vs. (**B**) OPLS-DA score plots of THP-1 cells. The figures show a clear separation between control, pooled and treatment groups (LPS, melittin and LPS + melittin) based on 128 significant non-polar metabolites separated on an ACE C4 column (*n* = 4). PCA score plot (**A**) has R^2^X = 0.867, Q^2^ = 0.753. OPLS-DA score plot (**B**) has R^2^X = 0.896, R^2^Y = 0.978, Q^2^ = 0.895. (C: control; Mel: melittin; LPS: lipopolysaccharide; Mel + LPS: melittin and LPS combination treatments; *P* = pooled samples).

Based on previous observations on the effect of melittin on cell lipids [24], other data sets were obtained using a reversed phase (RP) column (ACE C4 column) to characterise the changes in non-polar metabolites. As shown in Figure 7, the separation between groups was clearer for lipophilic metabolites than for polar metabolites using an OPLS-DA model. Clustering of pooled (P 1–3) samples in the PCA model showed the validity of the analysis and the stability of the instrument throughout the run.

Univariate comparisons of the changes in metabolites in each group are shown in Table 1 and demonstrate clear differences in the metabolite levels between cells treated with LPS and melittin alone, thereby confirming distinct metabolic profiles for the combination treatments (melittin + LPS). Several metabolomics pathways were significantly altered (particularly glycolysis, TCA cycle, OXPHOS, arginine and proline metabolism, and nucleotide metabolism). Clearly significant increases were noted in the levels of fatty acids, whereas glycerophospholipid levels were decreased by melittin regardless of the presence or absence of LPS.

**Figure 8:**
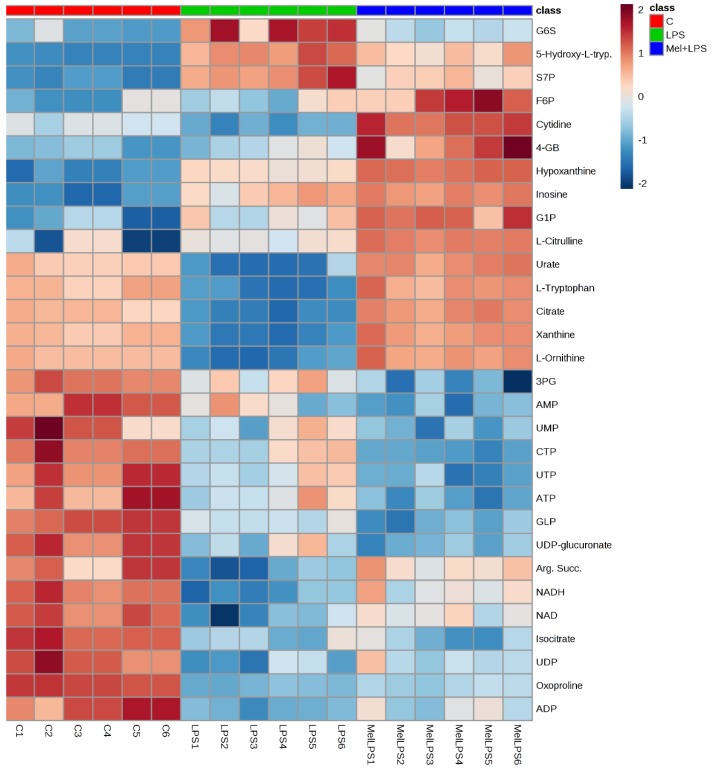
Heat map showing the top 30 significant putative metabolites among control (C), LPS, and melittin + LPS (Mel + LPS) using one-way ANOVA. The data are displayed on a log_2_ scale. The differences in colour shades represent intensities of the metabolites vs. sample observations.

The plot shows heat map of the top 30 significant metabolites from Table 1. The clearest effect in the heat map was that the similarity in the effect between LPS alone and Mel + LPS. Most of the metabolites were decreased with LPS treatment. Some metabolites are more abundant in combination treatment of Mel + LPS as compared to LPS alone, such as citrulline, hypoxanthine, and inosine (Figure 8).

## 4. Discussion

The efficacy of vaccines can be improved by the safe use of adjuvant substances, which are substances that modulate and enhance the immunogenicity of a vaccine antigen [53]. The aim of the present study was to investigate the potential usefulness of the BV compound, melittin as a natural vaccine adjuvant to stimulate the innate and adaptive immune systems. The research strategy was to characterise the ability of melittin to modify cytokine production in LPS-stimulated PMA-differentiated THP-1 cells. The effects of melittin, in combination with LPS, on the metabolic profiles of monocyte-derived macrophages were also evaluated to determine if metabolomic profiling of immune-modulating agents can provide a more in-depth understanding of the mechanisms and pathways of cytokine secretion.

The cytotoxicity assays showed that the THP-1 monocytes were no longer viable when exposed to 6.25 µg/mL melittin, while the PMA-differentiated cells retained 18% cell viability. No significant cell lysis was observed below 1.56 µg/mL for the normal THP-1 cells, which confirmed a greater melittin sensitivity of the cells with a lower IC_50_ value, when compared to cells differentiated into macrophages. The pro-inflammatory IL-1β showed the most enhanced release in response to melittin and the response was dose-dependent and also its release was significantly higher than LPS alone when 1 µg/mL of melittin was used in combination with LPS. A significant enhancement was also observed for IL-6 cytokine comparing with LPS alone with 0.5 µg/mL of melittin, but only a slight non-significant enhancement was observed for TNF-α which reflects the findings of an earlier study. Interestingly, the production of the anti-inflammatory IL-10 was decreased by melittin in the combination treatment with LPS, which might support the use of melittin as a pro-inflammatory enhancer. These results support our previous findings on the effect of melittin on cytokine secretion by the U937 cell line [34], using the same experimental design.

IL-1β, a prototypic pro-inflammatory cytokine, has an essential role in immune responses and possesses adjuvant activity [54,55]. The production of IL-1β and IL-18 is increased by aluminium hydroxide, a routinely used adjuvant, which is believed to activate caspase-1 [56]. Several studies have reported that melittin has adjuvant activity that may arise by a different mechanism, perhaps by enhancing the absorption properties of vaccines when they are administered nasally [36]. Melittin can serve as a mucosal adjuvant for antigens administered via the nasal route as it enhances antibody titres in a dose-dependent manner when conjugated with tetanus and diphtheria toxoids [37]. Another study has shown that melittin induces the synthesis of TNF-α and IL-1β in a time- and dose-dependent manner through activation of phospholipase A2 (PLA2) [57,58]. A deeper understanding of the immune-stimulatory mechanism of adjuvants is a prerequisite for the design of more sophisticated vaccines.

The comparisons of control cells versus LPS-, melittin-, and LPS + melittin-treated cells revealed large numbers of metabolite differences. Overall, a synergistic effect was clearly evident in terms of the ratios of some of the inflammatory response metabolites when comparing the combination treatment with LPS alone (Table 1). The LPS-stimulated macrophages showed upregulation of the arginine metabolism, which resulted in the increased production of citrulline, which is an indicator of increased NO production, a hallmark of inflammation [59]. The levels of citrulline were increased by melittin alone and there was a clear synergic effect in combination with LPS. The levels of arginine, a precursor of NO and citrulline, and the N-(L-arginino)-succinate level were decreased in response to LPS alone and melittin with LPS treatments, but the N-(L-arginino)-succinate was not depleted when melittin alone was used. This might be due to the increased expression of argininosuccinate synthase (Ass1) and argininosuccinate lyase (Asl) [60]. These two enzymes are used to maintain levels of arginine with arginine succinate being exported from the cells and then retrieved again and converted to arginine when the arginine level is depleted in order to optimize the production of NO [60]. NO nitrosylation inhibits the components of the electron transport chain, thereby leading to OXPHOS inhibition through the suppression of complex II [61].

Several studies have shown an increase in aerobic glycolysis and a decrease in the TCA cycle and electron transport chain in macrophages due to increased release of NO [62,63]. Decreases in TCA cycle activity will lead to increases in the production of mitochondrial reactive oxygen species (ROS) and subsequent cytokine secretion [13,61]. The subsequent decrease in ATP production (Table 1) then leads to reprogramming of the cell metabolites and switching to glycolysis after the decline in OXPHOS. This serves as a survival response to maintain the level of ATP in the cells [64]. Despite the small effect seen on acetyl-CoA, the LPS and combination treatments caused decreases of ~30% and ~20%, respectively, which confirmed the inhibition of the TCA cycle and diversion of pyruvate to lactate instead of acetyl-CoA [4,10]. At the same time, shunting glucose through the pentose phosphate pathway (PPP) generates nicotinamide adenine dinucleotide phosphate (NADPH), which showed a significantly increased level in response to LPS treatment alone and is required for oxidative reactions, glutathione recycling, NO production, and ROS production [1]. Melittin was reported to increase the production of ROS and to induce apoptosis in TNF-related apoptosis-inducing ligand (TRAIL)-resistant HepG2 cells and the fall in NADPH with the melittin treatments may indicate increased consumption, this would fit with the higher levels of citrulline in the melittin treatments [65].

An elevation in PPP metabolites can boost the production of purines and pyrimidines for biosynthesis in activated cells [66]. Knockdown of the carbohydrate kinase-like protein (CARKL), a sedoheptulose kinase that catalyses the production of D-sedoheptulose 7-phosphate (S7P), was reported after LPS treatment and could potentiate the production of cytokines and M1 macrophage polarisation [11,67]. An increased flow of glycolytic intermediate metabolites into the oxidative arm of the PPP generated more NADPH from the conversion of glucose-6-phosphate to ribulose-5-phosphate (Ru5P). When the cellular need for NADPH exceeded that required for biosynthesis of nucleotides, Ru5P passed into the redox arm of PPP to increase the flux to glycolysis by generating fructose-6-phosphate (F6P) and glucose-6-phosphate (G6P). Thus, F6P was strongly elevated in response to the melittin alone and melittin and LPS combination treatment. S7P, a key control metabolite, was particularly elevated. In the same study, metabolomics analysis to revealed the metabolites linked to CARKL expression demonstrated that ribose-5-phosphate, xyulose-5-phosphate, S7P, glyceraldehyde-3-phosphate (G3P), G6P, and F6P were elevated concomitantly with a marked depletion of some TCA metabolites (such as malate and fumarate) and decreases in NADH levels [11]. These findings are similar to the current metabolomics results of some metabolites including fumarate, Ru5P, S7P and F6P (Table 1).

In the present study, a strong effect on cellular purine and pyrimidine metabolism was observed for the combination treatment. Guanine, guanosine, inosine, urate, hypoxanthine, xanthine, xanthosine, adenine, AMP, cytosine, cytidine, CTP, UTP, and UMP all showed a clear synergistic effect for the combination treatment when compared with LPS alone (Table 1). The effect of melittin in lowering UMP, UDP, UTP and UDP–glucose is of interest since UDP–sugar conjugates are involved in protein N-glycosylation and are upregulated in M2 macrophages [59]. It has been proposed that melittin can reduce the number of M2-like macrophages in an increasing cell population, producing a relative increase in M1 macrophages [52]. A reduction in the population of M2 cells has also been linked to a lowering of glutamine catabolism, which was observed in the current case for the melittin LPS combination. These findings resemble those of a previous study that showed elevated levels of xanthine, hypoxanthine, inosine, guanine, and guanosine in gingival crevicular fluid inflammation sites when compared with a healthy human oral cavity [68]. Higher levels of hypoxanthine were also observed in response to macrophage activation by LPS in the RAW 264.7 cell line [41]. The hyperactivity of purine degradation and the increase in hypoxanthine (33-fold) and inosine (6-fold) in the melittin + LPS-treated cells represent potential biomarkers for inflammation, as these result in greater production of ROS through xanthine oxidase, thereby causing cellular oxidative stress [68]. These results support previous findings in patients with inflammatory arthritis, where the levels of hypoxanthine, xanthine, and urate were higher in synovial fluid than in plasma, and hypoxanthine concentrations were significantly higher in the plasma from patients than from the control group [69]. Metabolic profiling of rheumatoid arthritis patients also indicated a significant increase in hypoxanthine levels when compared to healthy controls [70]. Overall, the results in the present study indicated a significant change in amino acid and nucleotide biosynthesis upon activation by the combination of LPS and melittin. Cell reprogramming was clearly observed by the combination treatment, which supports the use of melittin to stimulate cellular immune responses and its potential use as an immune adjuvant agent.

Metabolic profiling of lipophilic metabolites separated using a reversed phase column revealed significant changes in fatty acids of interest. LPS caused a slight increase in fatty acid synthesis by increasing the expression of the mitochondrial citrate carrier [71]. This would cause an accumulation of citrate in the cell cytoplasm through exchange with malate, and the citrate is then converted to oxaloacetate and acetyl-CoA, using ATP. Acetyl-CoA is then converted to malonyl-CoA, which is used for fatty acid synthesis [4,72]. LPS treatment was also found to inhibit the activity of the AMP-kinase in macrophages, causing a decrease in β-oxidation of fatty acids to produce inflammatory mediators [20]. Melittin, alone or in combination with LPS, caused a significant increase in the level of several fatty acids. These increases would explain the ability of melittin to cause disruption of the cell membrane, as confirmed by several previous findings [25,73]. Arachidonic acid (i.e., eicosatetraenoic acid) release from the cells was significantly increased by 46-fold and 42-fold by treatment with melittin and melittin + LPS, respectively (Table 1 and Figure S15). Several studies have shown that melittin activates the arachidonic acid signalling pathway in many cell lines, including PC12 and L1210 [74,75], and in neurons [76]. The mechanism of arachidonic acid production is argued to occur through multiple pathways to regulate cellular activity. One possibility is that it works through the stimulation of phospholipase A_2_; however, this effect was reported to lack selectivity for phospholipase A2 and fatty acid release [74]. The production of linoleic acid and linolenic acid, a precursor of arachidonic acid, was significantly increased in response to melittin, which would support the lytic activity of melittin on the cell membrane and its ability to penetrate phospholipid bilayers [21].

Arachidonic acid has a considerable importance in the immune system and in the promotion of inflammation. It can be metabolised via enzymatic reactions, including cyclooxygenase (COX), lipoxygenase, cytochrome p450 (CYP 450), and the enzymes of the anandamide pathways to create 20-carbon molecules (eicosanoids) such as prostaglandin (PG) and other signalling molecules. Eicosanoids are produced mainly following stimulation of the inflammatory process and in innate immunity regulation [77,78]. Melittin was indicated to induce mRNA levels of COX-2 and IL-8 in fibroblast-like synoviocytes [21]. The apparent strong involvement of the arachidonic acid pathway in the production of pro-inflammatory mediators supports the potential use of melittin for immune activation and as an immune-adjuvant vaccine agent. In addition to the synergistic effect of melittin to LPS in most of the amino acids and nucleotides, its effect on cells lipids was very marked, suggesting membrane re-modelling of macrophages cells upon activation by melittin with a particular selectivity for lipids carrying long-chain polyunsaturated fatty acids.

## 5. Conclusions

In conclusion, PMA-differentiated THP-1 cells showed a significant enhancement in the secretion of IL-1β and IL-6 cytokines in response to treatment with a combination of melittin and LPS when compared to LPS alone when a 1 µg/mL concentration was used. Melittin combined with LPS also produced a decrease in the level of the anti-inflammatory IL-10 cytokines. However, there was little effect of melittin on the level of TNF-α, which mirrors what was observed previously. In addition, this study provides an outline of the metabolites associated with cytokine production and immune system activation by LPS, melittin, or LPS + melittin. The clearest effects of the combination of LPS and melittin treatment were on glycolysis, the TCA cycle, OXPHOS, and purine, pyrimidine, and fatty acid metabolism. The most marked effects were on the levels of polyunsaturated fatty acid and associated with this a reduction in the levels of some long-chain highly unsaturated phospholipids. Exposure to the combination treatment resulted in marked elevation of most fatty acids, and arachidonic acid in particular, suggesting different metabolic responses to LPS, melittin, and the melittin and LPS combination. The effect of melittin in lowering UTP levels and UDP-glucose fits with a previous report that UDP–sugar conjugates may be important for conferring M2-like properties to macrophages. To the authors’ knowledge, this is the first time that melittin has been shown to work as an immune adjuvant based on results from metabolic profiling of monocyte-derived macrophages. Our results emphasise that the use of melittin in combination with LPS could result in improved therapy for vaccine immunisation. Certainly, melittin shows promise as a potential vaccine adjuvant.

## Figures and Tables

**Table 1:** Significantly changed metabolites within THP-1 cells treated with lipopolysaccharide (LPS), melittin, and melittin + LPS in comparison with untreated controls. The results contain the majority of affected metabolites. (0.5 µg/mL LPS; 1 µg/mL melittin).

Mass	Rt	Putative Metabolite	LPS		Mel		Mel + LPS	
			Ratio	*p* Value	Ratio	*p* Value	Ratio	*p* Value
**Arginine and proline metabolism**								
175.096	15.25	L-Citrulline *	1.825	0.048	7.482	0.001	8.657	0.001
174.112	26.50	L-Arginine *	0.255	<0.001	1.111	ns	0.916	ns
240.122	15.55	Homocarnosine	0.640	0.005	1.570	0.002	1.481	0.001
145.085	14.52	4-Guanidinobutanoate	1.122	0.014	0.792	<0.001	1.611	<0.001
246.133	13.55	N2-(D-1-Carboxyethyl)-l-arginine	0.295	<0.001	0.951	ns	1.544	<0.001
113.059	9.30	Creatinine	0.288	<0.001	1.045	ns	1.234	ns
129.043	13.62	Oxoproline	0.135	<0.001	0.192	<0.001	0.182	<0.001
290.123	15.83	N-(L-Arginino) succinate	0.562	<0.001	1.367	0.006	0.832	0.040
132.090	23.04	L-Ornithine *	0.387	<0.001	1.051	ns	1.177	0.004
147.053	13.45	L-Glutamate *	0.661	<0.001	0.917	ns	0.855	0.026
398.137	16.01	S-Adenosyl-l-methionine	0.768	ns	5.717	<0.001	4.267	<0.001
115.063	12.14	L-Proline *	0.439	<0.001	0.909	ns	1.103	ns
**Glycolysis/TCA cycle**								
339.996	17.18	D-Fructose 1,6-bisphosphate *	1.397	ns	0.544	<0.001	0.671	0.009
260.030	15.02	D-Fructose 6-phosphate *	1.210	ns	1.620	<0.001	1.590	<0.001
260.020	15.94	D-Glucose 6-sulfate	2.193	0.000	1.359	ns	1.190	0.018
260.030	15.83	D-Glucose 1-phosphate *	1.366	0.004	2.264	<0.001	1.909	<0.001
809.125	12.25	Acetyl-CoA	0.713	0.027	0.946	ns	0.792	0.028
185.993	15.94	3-Phospho-D-glycerate	0.753	0.001	0.610	<0.001	0.501	<0.001
169.998	15.10	Glycerone phosphate	0.381	<0.001	0.315	<0.001	0.249	<0.001
192.027	17.28	Citrate *	0.318	<0.001	1.089	ns	1.297	0.001
192.027	18.47	Isocitrate *	0.489	<0.001	0.580	<0.001	0.466	<0.001
116.011	14.82	Fumarate *	0.751	0.013	0.891	ns	0.677	0.001
665.125	13.23	NADH *	0.324	<0.001	0.492	<0.001	0.584	<0.001
663.109	14.17	NAD+ *	0.482	<0.001	0.923	ns	0.671	<0.001
**OXPHOS/Pentose phosphate pathway**								
506.996	15.76	ATP *	0.751	0.006	0.987	ns	0.585	<0.001
427.030	14.31	ADP *	0.534	<0.001	0.812	0.012	0.640	<0.001
258.014	16.69	D-Glucono-1,5-lactone 6-phosphate	1.214	ns	1.207	ns	1.407	0.026
290.041	15.23	D-Sedoheptulose 7-phosphate	1.831	<0.001	1.181	0.002	1.489	<0.001
230.019	14.60	D-Ribulose 5-phosphate	0.932	ns	0.579	0.000	0.713	0.005
370.007	17.42	D-Sedoheptulose 1,7-bisphosphate	1.725	0.002	0.758	ns	1.065	ns
745.091	16.74	NADPH *	1.845	0.049	1.521	ns	0.508	0.048
743.075	16.51	NADP+	0.693	0.030	0.764	0.031	0.820	0.047
**Purine metabolism**								
151.049	11.82	Guanine *	1.300	ns	0.632	0.010	1.707	0.001
283.092	12.93	Guanosine	1.310	ns	0.619	0.015	2.185	0.001
348.047	14.40	IMP *	2.093	ns	2.305	0.021	1.727	ns
268.081	10.32	Inosine *	4.079	<0.001	1.852	0.001	6.051	<0.001
168.028	11.38	Urate *	0.168	<0.001	1.372	0.001	1.838	<0.001
136.039	9.74	Hypoxanthine *	7.345	<0.001	4.852	<0.001	33.584	<0.001
152.034	10.46	Xanthine	0.283	<0.001	1.109	0.043	1.264	0.003
284.076	9.72	Xanthosine	0.270	<0.001	1.019	ns	1.259	0.015
135.054	9.22	Adenine	1.246	ns	2.237	0.009	1.649	ns
347.063	12.62	AMP *	0.538	0.002	0.873	ns	0.339	<0.001
**Pyrimidine metabolism**								
403.018	16.15	CDP *	0.584	<0.001	0.690	0.001	0.650	0.002
111.043	11.38	Cytosine	0.684	ns	2.434	ns	3.660	0.021
482.985	17.60	CTP *	0.659	0.001	0.719	0.001	0.463	<0.001
243.086	11.36	Cytidine *	0.528	<0.001	2.375	<0.001	3.628	<0.001
483.969	17.02	UTP *	0.617	<0.001	0.685	<0.001	0.437	<0.001
324.036	15.17	UMP *	0.721	0.011	0.630	<0.001	0.589	<0.001
404.003	15.58	UDP *	0.422	<0.001	0.666	0.001	0.534	<0.001
580.035	17.95	UDP-glucuronate	0.604	<0.001	0.560	<0.001	0.495	<0.001
566.055	16.00	UDP-glucose	0.637	0.000	0.555	0.000	0.457	<0.001
605.077	17.17	GDP-mannose	1.812	<0.001	1.996	<0.001	1.532	<0.001
**Fatty acids and metabolites ^C4^**								
306.256	12.45	Eicosatrienoic acid *	1.650	0.004	1.983	ns	1.977	<0.001
328.240	18.27	Docosahexaenoic acid *	0.346	ns	6.658	0.002	5.846	0.002
318.219	4.10	Leukotriene A4 or isomer	1.115	ns	1.469	ns	2.183	0.002
310.287	17.73	Eicosenoic acid *	1.328	ns	0.477	ns	0.313	0.009
356.256	14.78	Prostaglandin F1alpha or isomer	0.276	0.007	0.479	0.024	0.482	0.048
336.230	4.55	Prostaglandin B1 or isomer	1.004	ns	1.432	ns	2.125	<0.001
402.225	4.00	5S-HETE di-endoperoxide or isomer	1.709	ns	0.850	ns	0.650	0.007
282.256	20.06	Oleic acid *	1.239	0.036	9.007	<0.001	8.089	<0.001
256.240	7.84	Hexadecanoic acid isomer	1.397	0.005	7.235	<0.001	13.602	0.004
338.319	24.61	Docosenoic acid *	1.246	0.005	2.740	<0.001	2.941	<0.001
268.240	11.26	Heptadecenoic acid	1.362	0.013	1.782	<0.001	1.211	ns
366.350	26.59	Tetracosenoic acid *	1.354	0.038	2.665	<0.001	2.800	<0.001
304.240	18.36	Eeicosatetraenoic acid *	1.049	ns	46.370	<0.001	42.444	<0.001
332.272	20.30	Docosatetraenoic acid	0.929	ns	25.404	<0.001	21.474	<0.001
242.225	17.97	Pentadecanoic acid *	0.967	ns	1.787	<0.001	1.758	<0.001
334.287	21.82	Docosatrienoic acid *	0.776	ns	8.679	0.001	7.684	0.001
226.193	15.25	Tetradecenoic acid	0.890	ns	3.570	0.002	3.367	0.003
270.256	12.86	Heptadecanoic acid	0.874	ns	4.801	0.010	1.854	0.009
278.225	17.08	Linolenic acid *	0.789	0.029	3.716	<0.001	2.861	<0.001
280.240	18.38	Linoleate *	1.105	ns	9.760	<0.001	8.943	<0.001
**Glycerophospholipids ^C4^**								
721.467	12.69	PC 32:6	0.672	ns	0.214	0.016	0.217	0.023
832.654	30.65	PG 41:0 ether	0.665	0.028	0.623	0.019	0.402	0.003
738.448	12.65	PG34:6	0.949	ns	0.357	0.020	0.308	0.015
484.280	13.33	Lyso PG 16:0	1.589	ns	0.449	0.034	0.283	0.013
800.449	12.56	PI 32:5	0.885	ns	0.365	0.009	0.329	0.005
584.333	13.28	Lyso PI 18:0 Ether	0.941	ns	0.558	0.020	0.499	0.004
858.526	13.47	PI 36:4	1.841	ns	0.441	0.025	0.316	0.027
814.501	13.55	PI 34:4 ether	2.088	ns	0.486	0.025	0.317	0.019
785.521	32.15	PS 36:3	0.789	0.008	0.782	0.005	0.779	0.007
517.244	12.56	Lyso PS 18:4	1.184	ns	2.645	<0.001	1.659	0.022
833.520	22.16	PS 40:7	1.118	ns	0.439	0.004	0.470	0.009
777.457	13.50	PS 36:7	0.970	ns	0.432	0.023	0.371	0.010
**Miscellaneous**								
131.058	13.88	N-Acetyl-beta-alanine	2.017	0.004	4.879	0.001	5.950	0.001
217.143	15.88	beta-Alanyl-L-lysine	1.543	0.041	3.816	<0.001	1.705	0.021
182.079	13.10	D-Sorbitol *	0.325	<0.001	2.226	<0.001	4.443	<0.001
180.064	12.66	D-Mannose *	0.712	<0.001	1.538	<0.001	1.744	<0.001
180.064	14.19	D-Galactose	1.326	ns	3.785	0.001	4.184	0.001
221.090	11.16	N-Acetyl-D-glucosamine *	0.464	<0.001	0.953	ns	0.806	0.008
146.069	14.34	L-Glutamine *	0.370	<0.001	1.085	ns	1.322	0.002
129.043	10.45	5-Oxoproline *	2.424	0.018	0.897	ns	1.192	ns
220.085	8.96	5-Hydroxy-L-tryptophan *	7.211	<0.001	1.131	0.010	4.305	<0.001
204.090	11.14	L-Tryptophan *	0.190	<0.001	1.189	ns	1.311	0.046
181.074	12.34	L-Tyrosine *	0.309	<0.001	1.050	ns	1.265	0.042
131.095	10.77	L-Leucine *	0.217	0.008	1.038	ns	1.226	0.048
117.079	11.94	L-Valine *	1.615	0.001	6.492	0.000	2.822	0.002
612.152	16.40	Glutathione disulfide	0.652	<0.001	1.186	0.047	1.109	ns
142.074	13.04	Ectoine *	1.164	ns	0.553	<0.001	0.196	<0.001
131.069	13.99	Creatine *	0.547	<0.001	0.615	<0.001	0.439	ns
103.100	20.48	Choline *	0.444	<0.001	3.571	<0.001	2.827	<0.001
105.043	15.44	L-Serine *	1.518	<0.001	1.737	<0.001	1.707	<0.001
121.020	15.41	L-Cysteine *	0.581	<0.001	2.182	<0.001	4.862	<0.001
155.070	15.87	L-Histidine *	1.088	ns	2.089	<0.001	3.122	<0.001
161.105	12.59	L-Carnitine *	0.608	<0.001	0.822	0.008	0.744	0.002
238.230	12.66	2-trans-Hexadecenal ^C4^	0.795	ns	16.865	0.000	12.016	0.000

Rt: Retention time (min); LPS: Lipopolysaccharides; Mel: melittin; Mel + LPS: melittin + LPS combination treatment; C4: Detected by ACE C4 column; *: Matches the analytical standard retention time; ns: Non-significant.

## Supplementary Materials

The following are available online at http://www.mdpi.com/2076-393X/6/4/72/s1, Figure S1: Showing MPLC separation of BV components, Figure S2: Microscopic comparison between negative control THP-1 cells and its derived macrophages, Figure S3–S14: Showing a representative 4-parameter logistic plot of TNF-α, IL-1β, IL-6, and IL-10 standard samples, Figure S15: Extracted ion chromatograms for arachidonic acid, Table S1–S4: Effect of melittin on the production of TNF-α, IL-1β, IL-6, and IL-10 cytokines in the presence and absence of LPS, Table S5: List of abbreviation, Table S6: List of catalog/serial number of instruments and reagents.
