# Supplementary material for: Effect of Melittin on Metabolomic Profile and Cytokine Production in PMA-Differentiated THP-1 Cells
Source: Vaccines (Basel). 2018 Oct 13;6(4):72. doi: 10.3390/vaccines6040072 (PMC6313865; doi:10.3390/vaccines6040072)
Supplement: Supplementary file 1 [file vaccines-06-00072-s001.pdf]

Supplementary Materials: Effect of Melittin on Metabolomic Profiling and Cytokine Production in PMA-Differentiated THP-1 Cells

Abdulmalik Alqarni, Valerie A. Ferro, John A. Parkinson, Mark Dufton and David G. Watson

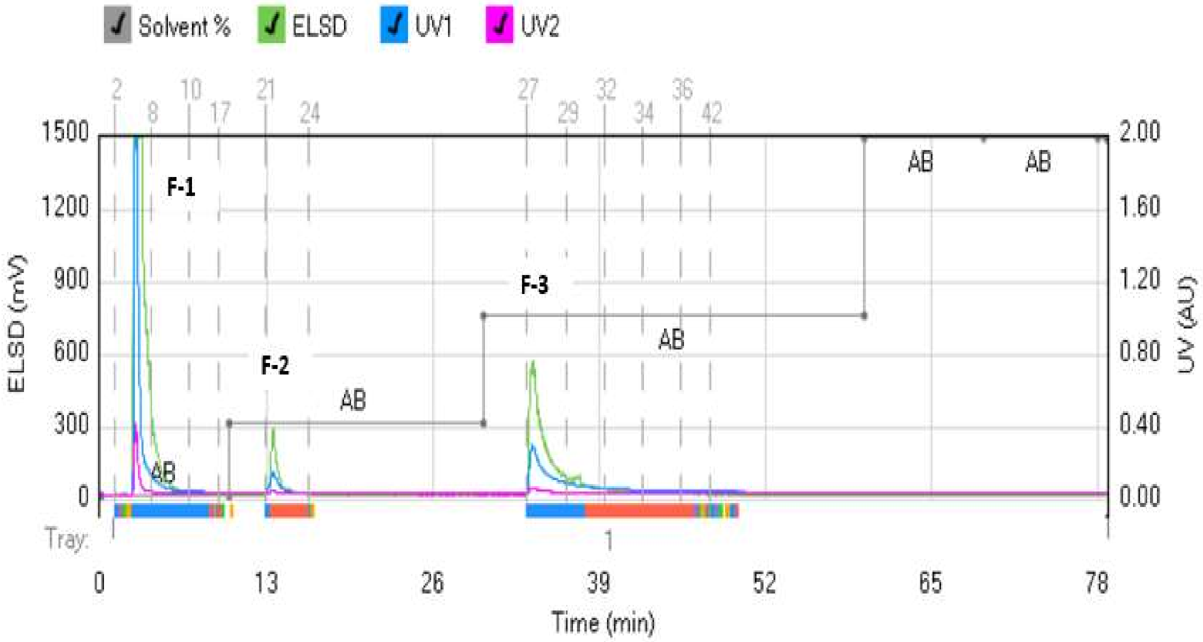

Figure S1: Chromatogram obtained from MPLC for the separation of BV components using the Grace® system. Generic C18, 24g column used; solvents: water (A) and acetonitrile (B) with a gradient of 0-10 min (0% B), 10-20 min (20% B), 20-30 min (50% B), 30-60 min (60% B), 60-70 min (100% B). The colours on the x-axis represent separate collections across with of the peak.

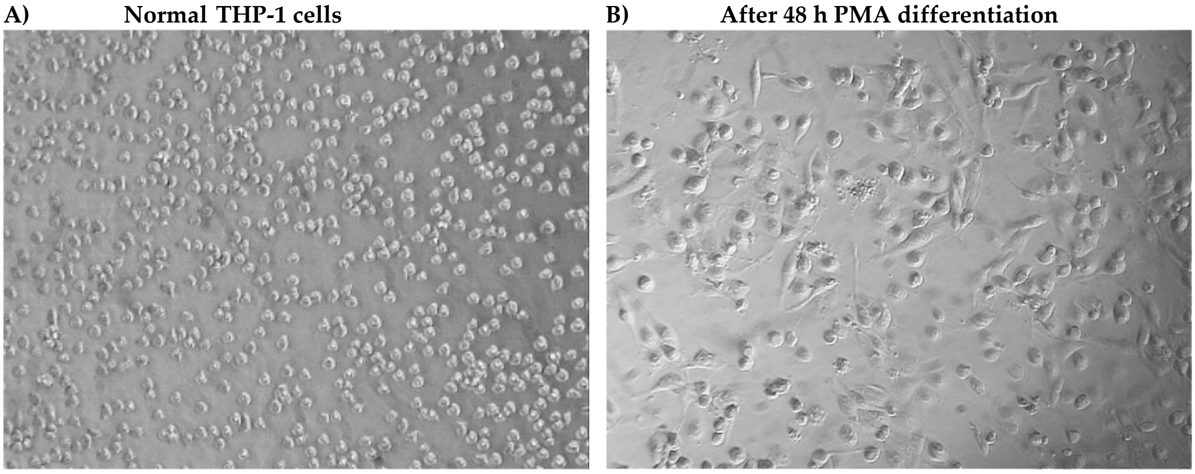

Figure S2: Negative control THP-1 cells **(A)** and it is derived macrophages by the effect of PMA treatment **(B)** at final concentration of 60ng/mL.

TNF-α production

Table S1: Effect of melittin (Mel) on the production of TNF-α cytokines in the presence and absence of LPS on PMA-differentiated THP-1 cells (*n*=3).

	TNF-α *concentration (pg/ml)*								
**Dose (µg/ml)**	**Media**	**LPS**		**Sample**		**Sample + 0.5 LPS**		**Sample + 1 LPS**	
		**0.5 LPS**	**1 LPS**	**0.5 Mel**	**1 Mel**	**0.5 Mel**	**1Mel**	**0.5 Mel**	**1 Mel**
**n=1**	458	1856	1862	315	381	1942	1879	1947	1885
**n=2**	736	1830	1848	833	752	1839	1881	1869	1825
**n=3**	561	1776	1847	482	722	1859	1826	1881	1862
**Mean**	585	1820.67	1852.3	543.33	618.33	1880	1862	1899	1857.33
**RSD**	24.02	2.24	0.45	48.66	33.33	2.91	1.68	2.21	1.63
***p*.value**	n/a	<0.001	<0.001	ns	ns	ns	ns	ns	ns

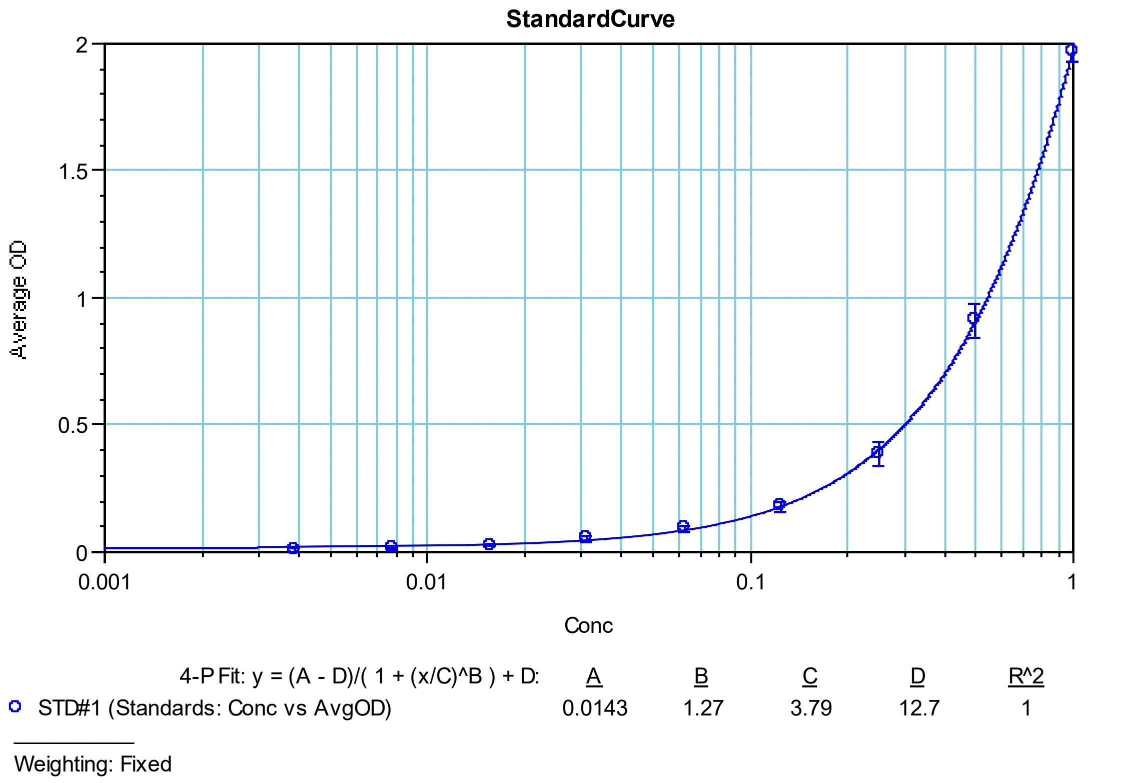

Figure S3: A representative 4-parameter logistic plot of TNF-α standard samples of 9 points showing the values of a, b, c, and d constants and the calibration equation with a perfect fit (R^2=1.0). The data represents the mean ± SD of optical density (OD) values for duplicate standard concentrations (*n*=2).

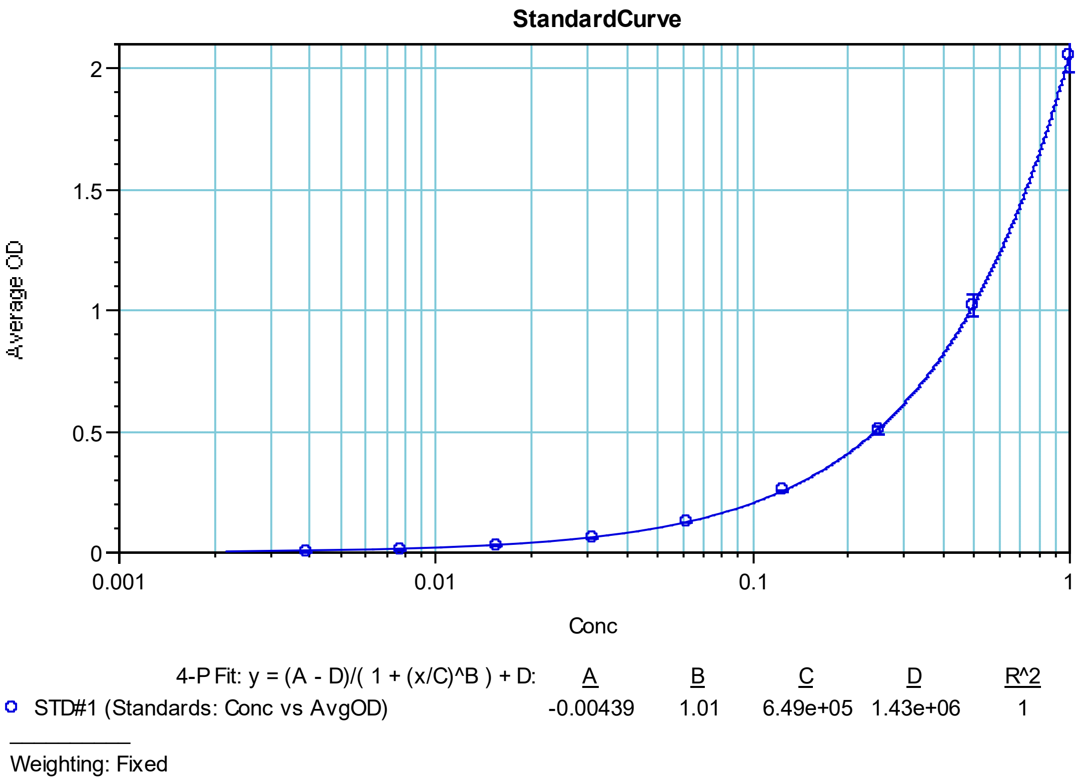

Figure S4: A representative 4-parameter logistic plot of TNF-α standard samples of 9 points showing the values of a, b, c, and d constants and the calibration equation with a perfect fit (R^2=1.0). The data represents the mean ± SD of optical density (OD) values for duplicate standard concentrations (*n*=2).

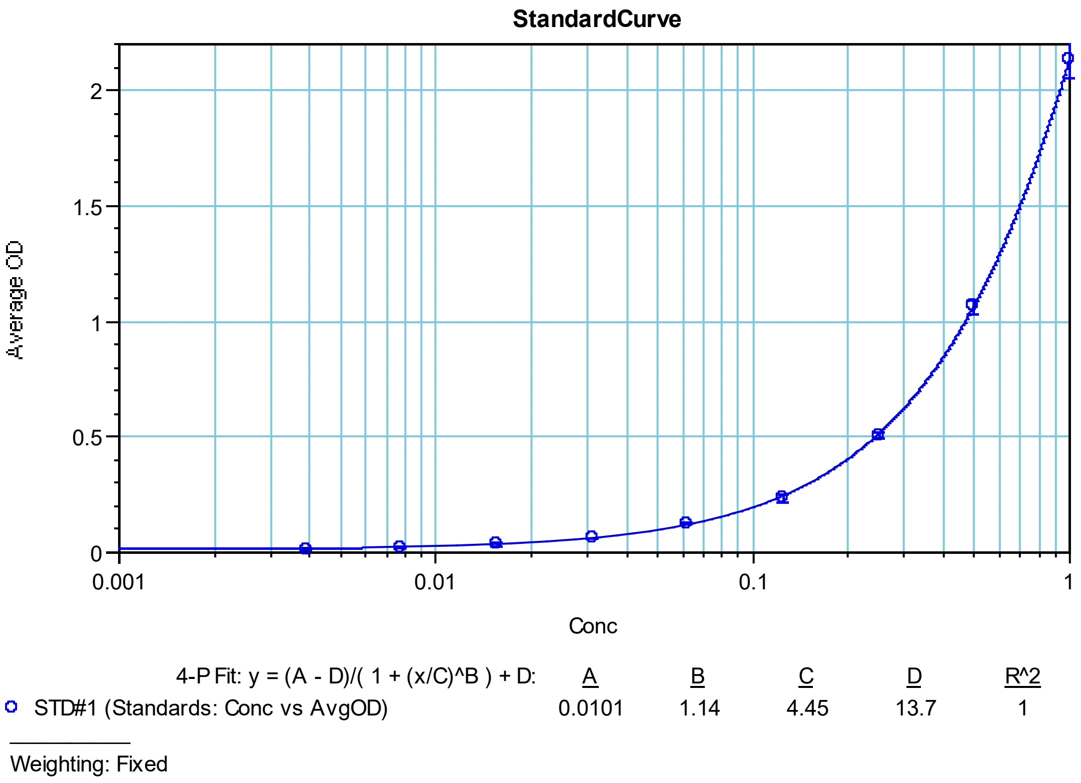

Figure S5: A representative 4-parameter logistic plot of TNF-*α* standard samples of 9 points showing the values of a, b, c, and d constants and the calibration equation with a perfect fit (R^2=1.0). The data represents the mean ± SD of optical density (OD) values for duplicate standard concentrations (*n*=2).

IL-1β production

Table S2: Effect of melittin (Mel) on the production of IL-1β cytokines in the presence and absence of LPS on PMA-differentiated THP-1 cells (*n*=3).

	**IL-1β *concentration (pg/ml)***								
**Dose (μg/ml)**	**Media**	**LPS**		**Sample**		**Sample+ 0.5 LPS**		**Sample + 1 LPS**	
		**0.5 LPS**	**1 LPS**	**0.5 Mel**	**1 Mel**	**0.5 Mel**	**1Mel**	**0.5 Mel**	**1Mel**
**n=1**	10.5	48.5	52	16	85	80	175	70	200
**n=2**	45	92	86	73	115	130	137	147	161
**n=3**	41	99	108	80	157	137	141	138	150
**Mean**	32.17	79.83	82.00	56.33	119.00	115.67	151	118.33	170.33
**RSD**	58.66	34.27	34.41	62.32	30.39	26.88	13.83	35.58	15.43
***p*.value**	n/a	ns	ns	ns	0.021	ns	0.026	ns	0.017

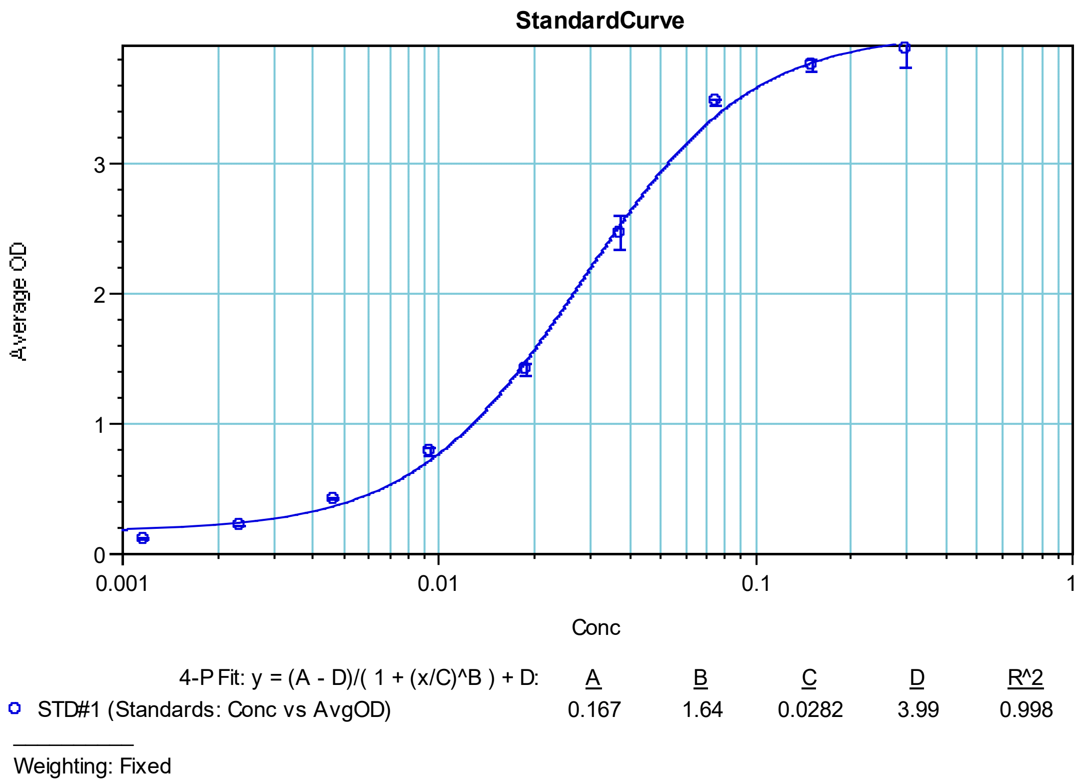

Figure S6: A representative 4-parameter logistic plot of IL-1β standard samples of 9 points showing the values of a, b, c, and d constants and the calibration equation with a good fit (R^2=0.998). The data represents the mean ± SD of optical density (OD) values for duplicate standard concentrations (*n*=2).

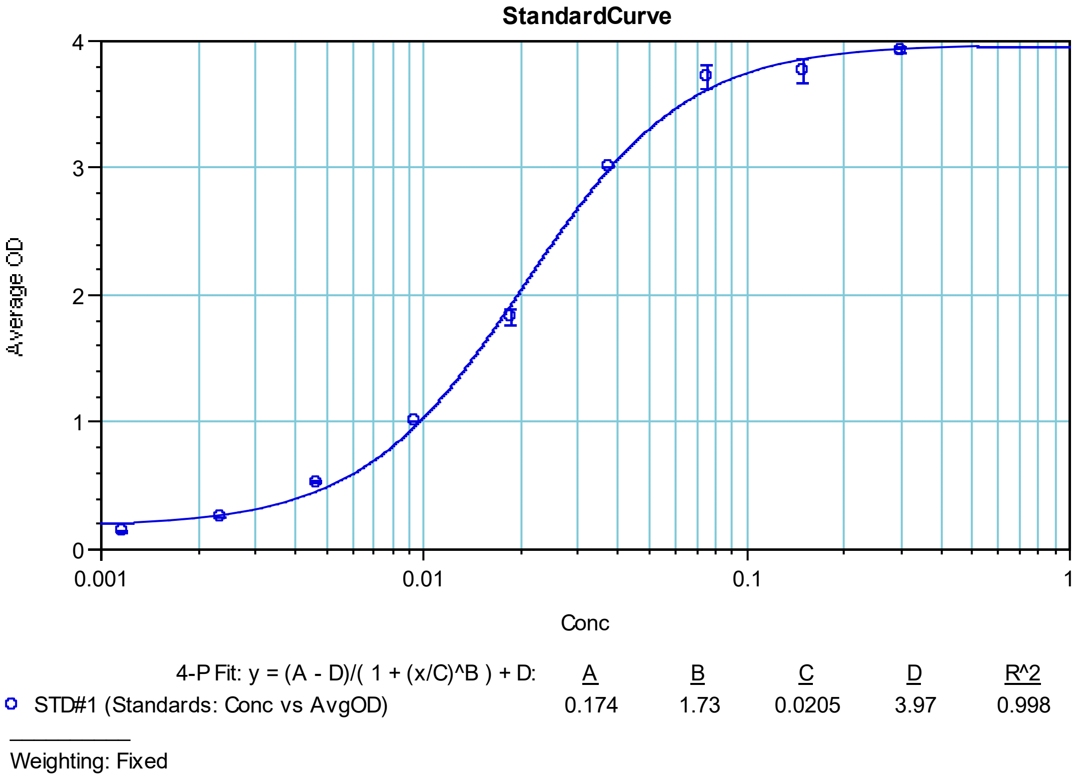

Figure S7: A representative 4-parameter logistic plot of IL-1β standard samples of 9 points showing the values of a, b, c, and d constants and the calibration equation with a good fit (R^2=0.998). The data represents the mean ± SD of optical density (OD) values for duplicate standard concentrations (*n*=2).

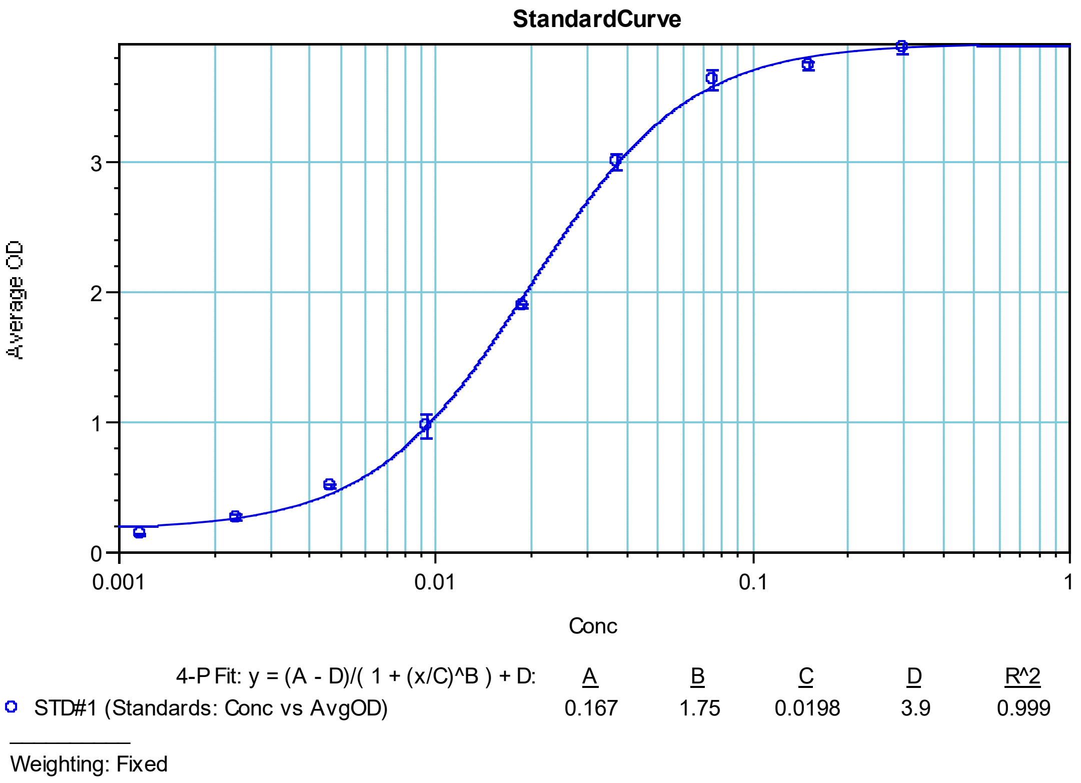

Figure S8: A representative 4-parameter logistic plot of IL-1β standard samples of 9 points showing the values of a, b, c, and d constants and the calibration equation with a perfect fit (R^2=0.999). The data represents the mean ± SD of optical density (OD) values for duplicate standard concentrations (*n*=2).

IL-6 production

Table S3: Effect of melittin (Mel) on the production of IL-6 cytokines in the presence and absence of LPS on PMA-differentiated THP-1 cells (*n*=3).

	**IL-6 *concentration (pg/ml)***					
**Dose (µg/ml)**	**Media**	**LPS**		**Sample**	**Sample + 0.5 LPS**	**Sample + 1 LPS**
		**0.5 LPS**	**1 LPS**	**0.5 Mel**	**0.5 Mel**	**0.5 Mel**
**n=1**	< 2.0	41	99	< 2.0	82	132
**n=2**	< 2.0	100	108	< 2.0	106	133
**n=3**	< 2.0	98	116	< 2.0	113	144
**Mean**	n/a	79.66	107.66	n/a	100.33	136.33
**RSD**	n/a	42.05	7.89	n/a	16.20	4.88
**P value**	n/a	n/a	n/a	n/a	ns	0.010

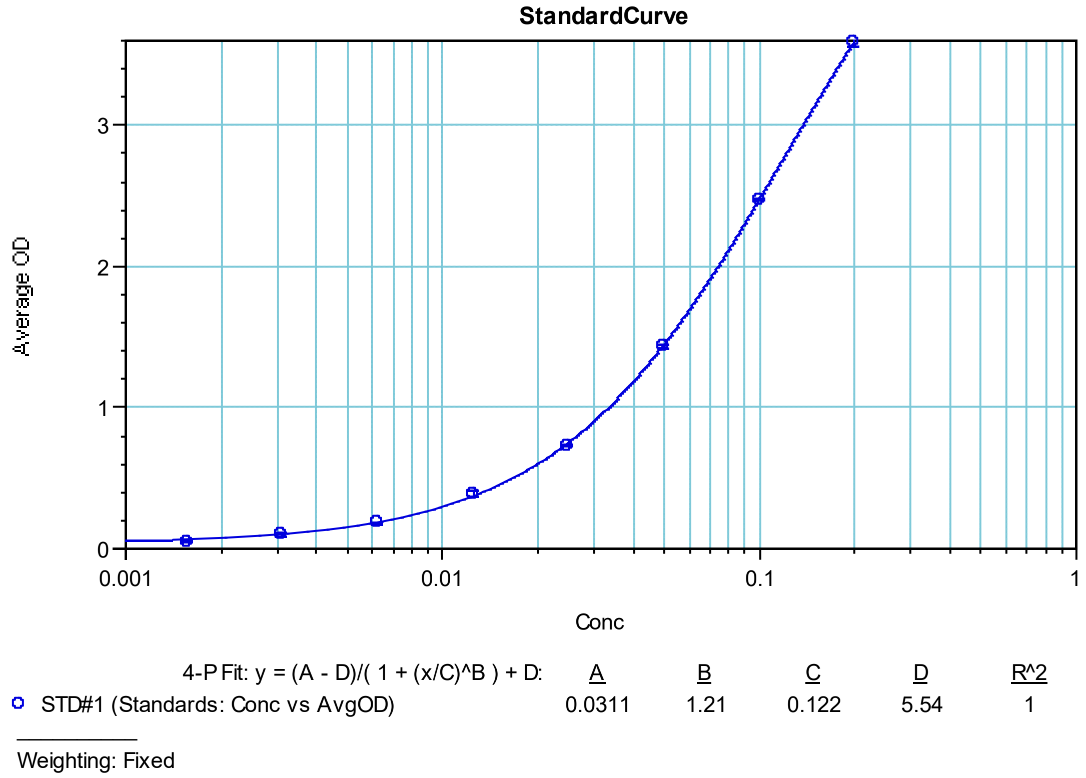

Figure S9: A representative 4-parameter logistic plot of IL-6 standard samples of 8 points showing the values of a, b, c, and d constants and the calibration equation with a perfect fit (R^2=1). The data represents the mean ± SD of optical density (OD) values for duplicate standard concentrations (*n*=2).

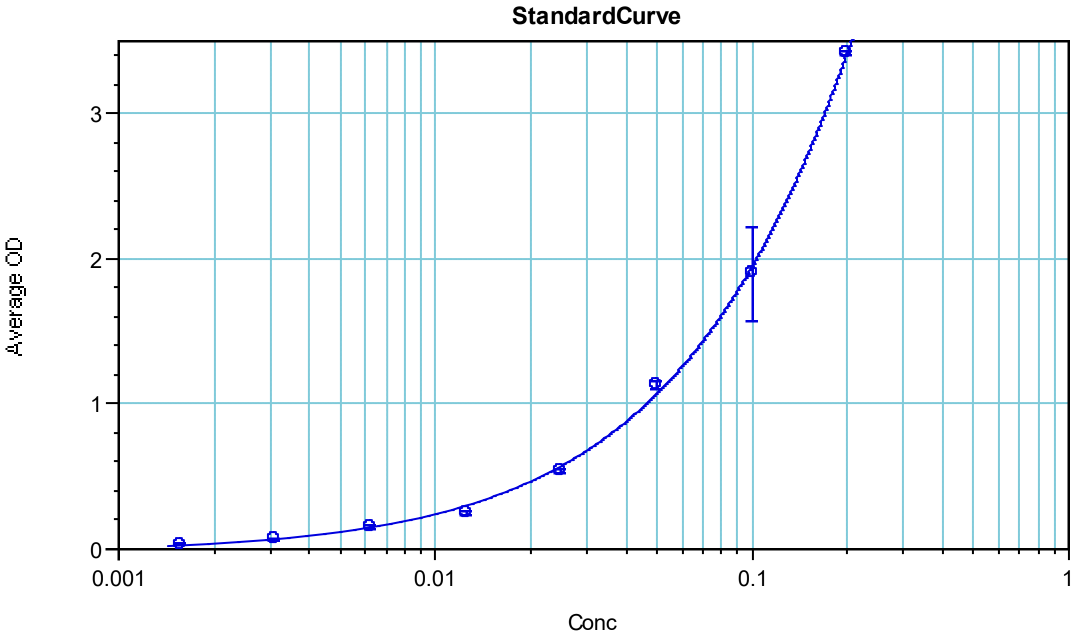

4-P Fit: y = (A - D)/(1 + (x/C)^B) + D:	A	B	C	D	R^2
STD#1 (Standards: Conc vs AvgOD)	-0.0324	0.902	1.56	25.4	0.999

Weighting: Fixed

Figure S10: A representative 4-parameter logistic plot of IL-6 standard samples of 8 points showing the values of a, b, c, and d constants and the calibration equation with a perfect fit (R^2=0.999). The data represents the mean ± SD of optical density (OD) values for duplicate standard concentrations (*n*=2).

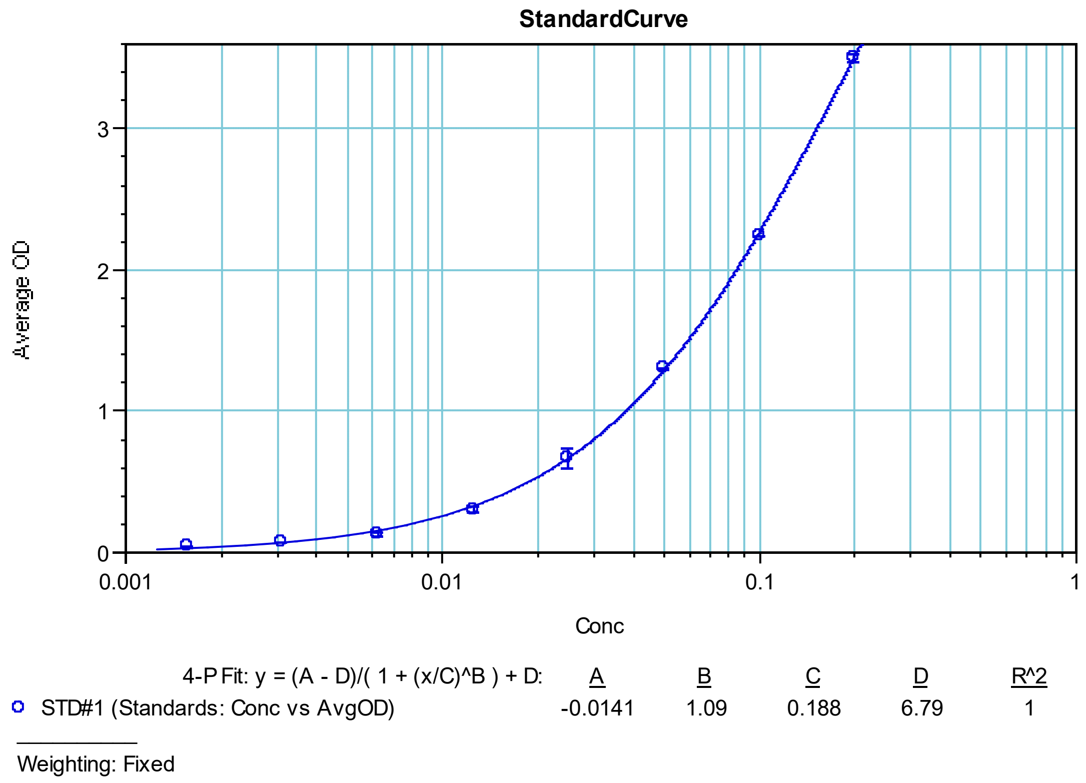

Figure S11: A representative 4-parameter logistic plot of IL-6 standard samples of 8 points showing the values of a, b, c, and d constants and the calibration equation with a perfect fit (R^2=1). The data represents the mean ± SD of optical density (OD) values for duplicate standard concentrations (*n*=2).

IL-10 production

Table S4: Effect of melittin (Mel) on the production of IL-10 cytokines in the presence and absence of LPS on PMA-differentiated THP-1 cells (*n*=3).

	**IL-10** ***concentration (pg/ml)***					
**Dose (µg/ml)**	**Media**	**LPS**		**Sample**	**Sample + 0.5 LPS**	**Sample + 1 LPS**
		**0.5 LPS**	**1 LPS**	**0.5 Mel**	**0.5 Mel**	**0.5 Mel**
**n=1**	27.5	62	52	24	56	57
**n=2**	17	30	41	12	32	33
**n=3**	20	40	40	20	26	34
**Mean**	21.50	44.00	44.33	18.67	38.00	41.33
**RSD**	25.16	37.21	15.02	32.73	41.78	32.85
**P value**	n/a	ns	0.010	ns	ns	ns

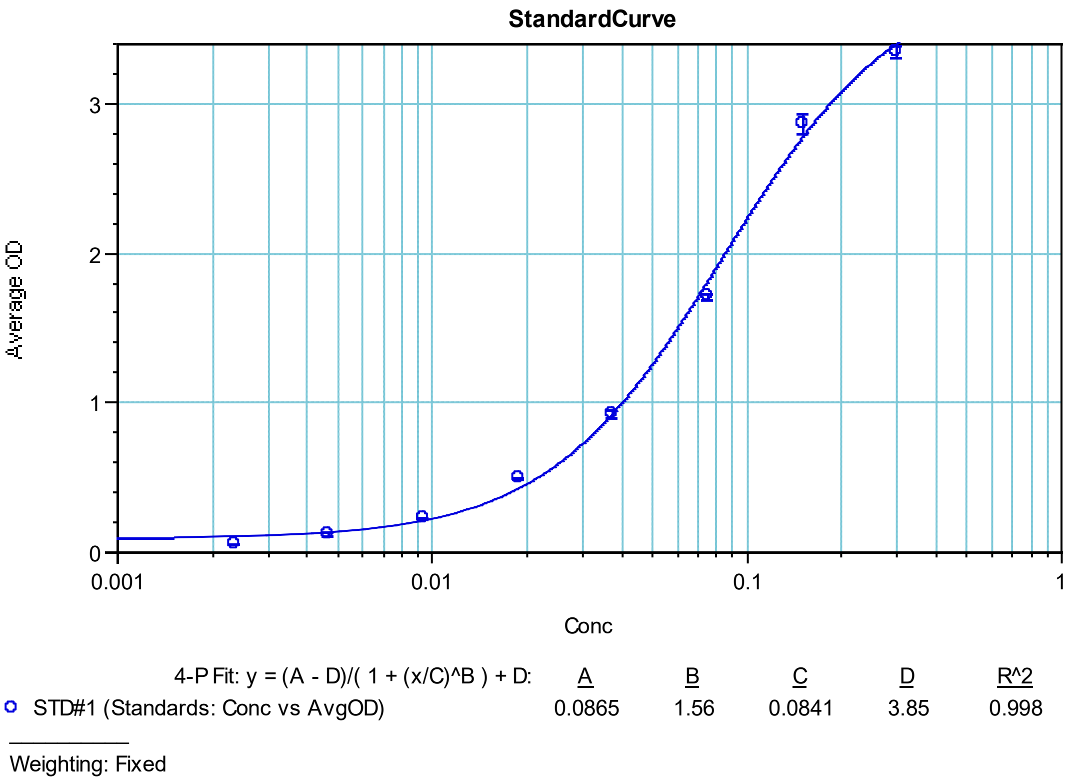

Figure S12: A representative 4-parameter logistic plot of IL-10 standard samples of 8 points showing the values of a, b, c, and d constants and the calibration equation with a good fit (R^2=0.998). The data represents the mean ± SD of optical density (OD) values for duplicate standard concentrations (*n*=2).

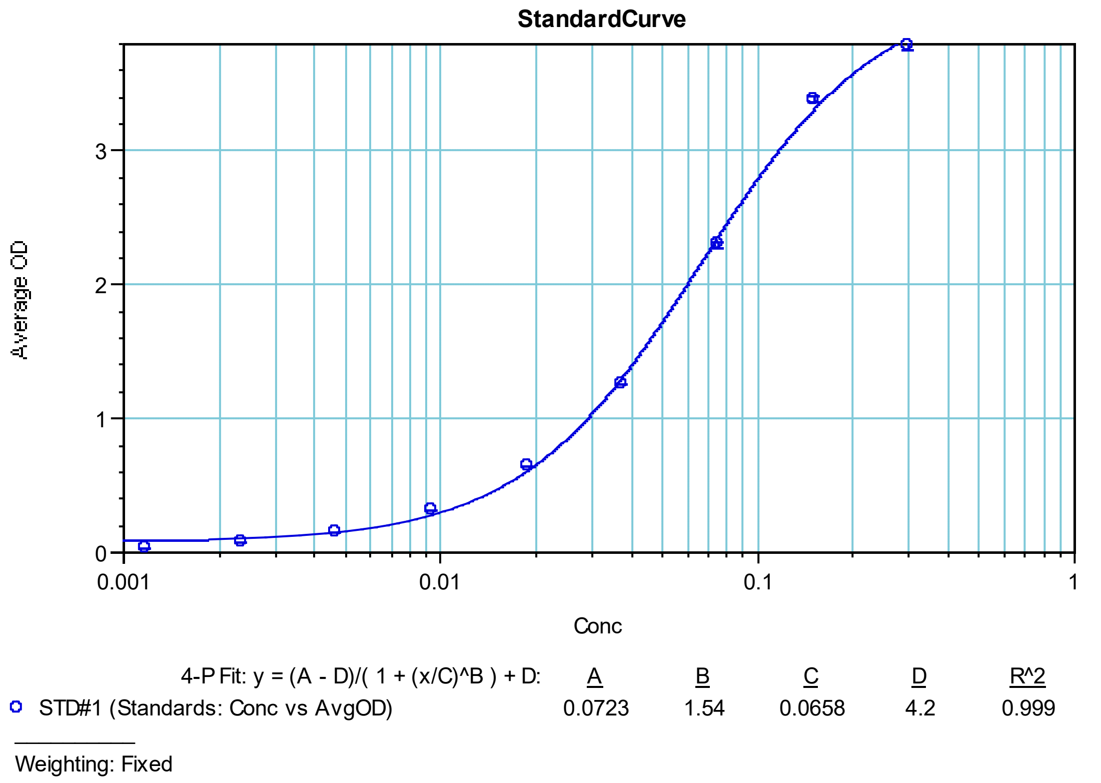

Figure S13: A representative 4-parameter logistic plot of IL-10 standard samples of 8 points showing the values of a, b, c, and d constants and the calibration equation with a perfect fit (R^2=0.999). The data represents the mean ± SD of optical density (OD) values for duplicate standard concentrations (*n*=2).

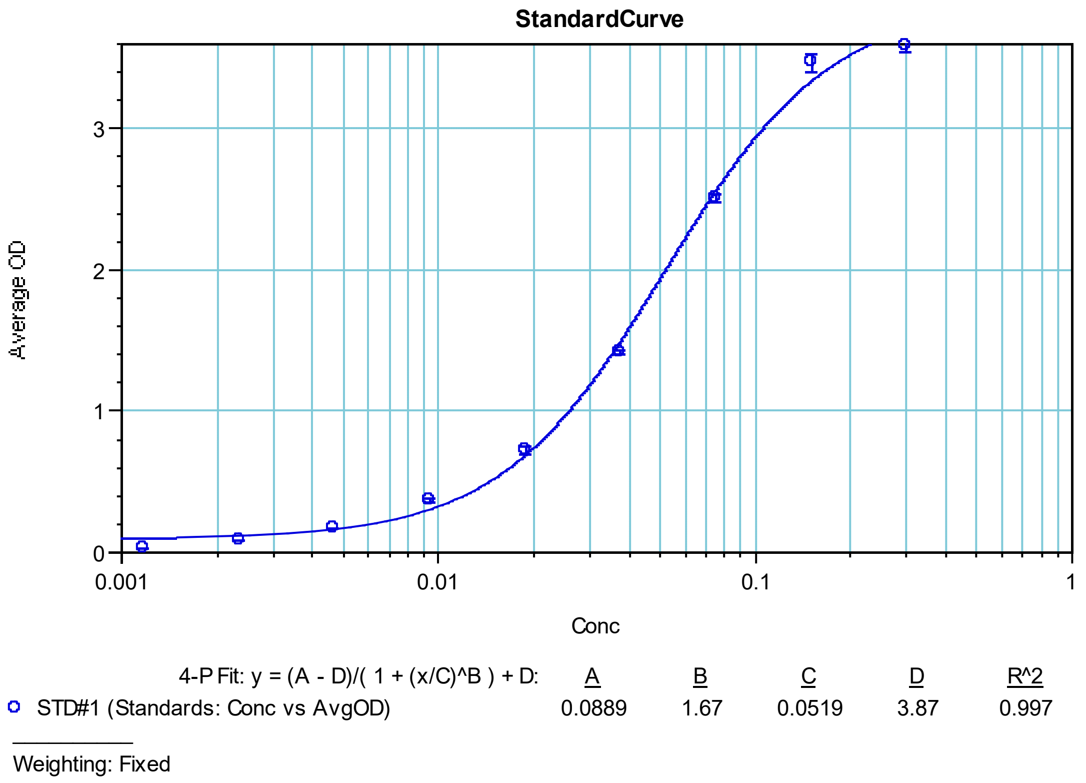

Figure S14: A representative 4-parameter logistic plot of IL-10 standard samples of 8 points showing the values of a, b, c, and d constants and the calibration equation with a good fit (R^2=0.997). The data represents the mean ± SD of optical density (OD) values for duplicate standard concentrations (*n*=2).

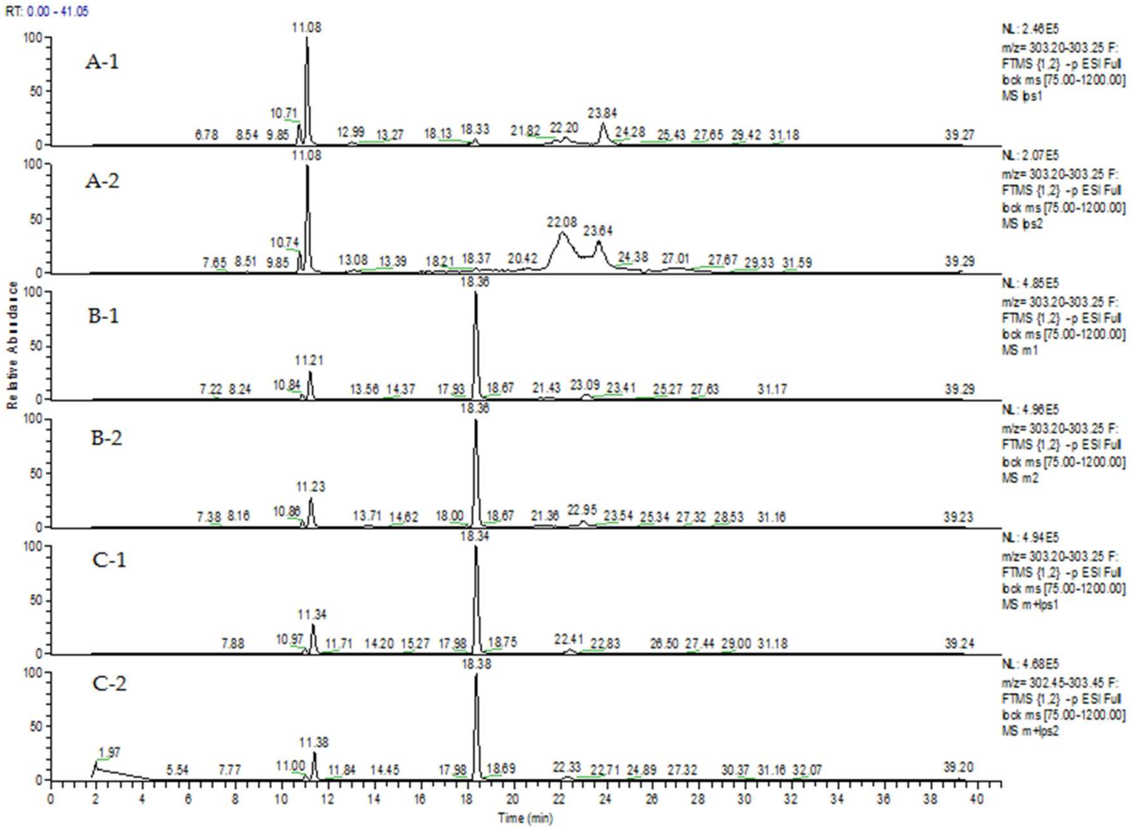

Figure S15: Extracted ion chromatograms for arachidonic acid in THP-1 cells after treated by LPS (A), melittin (B) and the combination of melittin and LPS (C). The level of the arachidonic acid elevated significantly by melittin alone or in combination with LPS. The biological samples were analysed using ACE C4 column.

Table S5: List of abbreviation used in this study.

**List of Abbreviations**	
HILIC	Hydrophilic Interaction Liquid Chromatography
RP	Reversed Phase
HPLC	High Performance Liquid Chromatography
LC-MS	liquid chromatography-mass spectrometry
UPLC-MS	Ultra-Performance Liquid Chromatograph- Mass Spectrometer
NMR	Nuclear Magnetic Resonance
ELISAs	Enzyme-linked immunosorbent assay
SIMCA	Soft-Independent Modelling of Class Analogy
OPLS-DA	Orthogonal Partial Least Squares Discriminant Analysis
PCA	Principal Component Analysis
QC	Quality control
RT	Retention Time
PLA2	Phospholipase A2
PBS	Phosphate Buffered Saline
KEGG	Kyoto Encyclopedia of Genes and Genomes
TCA	Cycle Tricarboxylic Acid cycle
OXPHOS	Oxidative phosphorylation
ATP	Adenosine Triphosphate
ADP	Adenosine Diphosphate

NAD+	Nicotinamide Adenine Dinucleotide (oxidised)
NADH	Nicotinamide Adenine Dinucleotide (reduced)
NADP+	Nicotinamide Adenine Dinucleotide phosphate (oxidised)
NADPH	Nicotinamide Adenine Dinucleotide phosphate (reduced)
F-2,6-BP	Fructose-2,6-bisphosphate
F6P	Fructose-6-phosphate
G6P	Glucose-6-phosphate
G3P	glyceraldehyde-3-phosphate
S7P	Sedoheptulose 7-phosphate
IMP	Inosine monophosphate
AMP	Adenosine monophosphate
CDP	Cytidine diphosphate
CTP	Cytidine Triphosphate
UTP	Uridine-5'-triphosphate
UDP	Uridine diphosphate
UMP	Uridine monophosphate
4-GB	4-Guanidinobutanoate
G6S	D-Glucose 6-sulfate
GLP	Glycerone phosphate
3PG	3-Phospho-D-glycerate
Arg. Succ.	N-(L-Arginino)succinate
Glu-1,6-L-6-P	D-Glucono-1,5-lactone 6-phosphate
5-Hydroxy-L-tryp.	5-Hydroxy-L-tryptophan
PMA	Phorbol 12-myristate 13-acetate
PC	Phosphocholines
PI	Phosphoinositol
PS	Phosphoserines
PG	Phosphoglycerols
LPS	Lipopolysaccharide
Mel	Melittin
PAMPs	Pathogen-associated molecular patterns
PRRs	Pattern Recognition Receptors
TLRs	Toll-like receptors
ROS	Reactive oxygen species
iNOS	Nitric oxide synthase
NO	Nitric oxide
HIF-1α	Hypoxia inducible factor-1α
5′TOP	5′-terminal oligopyrimidine
AMPK	Adenosine monophosphate-activated protein kinase
PFK2	Phosphofructokinase-2
mTOR	Mammalian target of rapamycin
MCD	Mast cell degranulating
BV	Bee venom
PGE2	Prostaglandin E2
Nuclear factor kappa B	NF-κB

Table S6: List of catalog/serial number of instruments and reagents used in this study.

Catalog/serial numbers	
HPLC	5035.0016
MS	SN01059P
Reveleris® iES system	1912L00078
plate reader	MV02120
ZIC-pHILIC column	543895
ACE C4 column	A73193
TNF- α ELISA Ready-Set-Go kits	88-7346-88
IL-1β ELISA Ready-Set-Go kits	88-7261-88

IL-6 ELISA Ready-Set-Go kits	88-7066-88
IL-10 ELISA Ready-Set-Go kits	88-7106-88
RPMI 1640 media	15-040-CVR
foetal calf serum	F13-1090/500
L-glutamine solution	RNBF8011
Penicillin/Streptomycin	015M4769V

© 2018 by the authors. Submitted for possible open access publication under the terms and conditions of the Creative Commons Attribution (CC BY) license (http://creativecommons.org/licenses/by/4.0/).
